# Process evaluation of an intervention developed using a participatory approach targeting school-related sedentary behaviour among adolescents

**DOI:** 10.1186/s12889-026-27847-7

**Published:** 2026-06-10

**Authors:** Veerle Van Oeckel, Benedicte Deforche, Janneke de Boer, Lea Rahel Delfmann, Teatske Altenburg, Femke De Meester, Maïté Verloigne

**Affiliations:** 1 Department of Public Health and Primary Care, Ghent University, Corneel Heymanslaan 10, Ghent, 9000 Belgium; 2 Department of Movement and Sport Sciences, Vrije Universiteit Brussel, Pleinlaan 2, Brussels, 1050 Belgium; 3 Department of Movement and Sports Sciences, Ghent University, Watersportlaan 2, Ghent, 9000 Belgium; 4 Diabetes Research Centre, College of Life Sciences, University of Leicester, Leicester, UK; 5 Leicester Diabetes Centre, Leicester General Hospital, University Hospitals of Leicester NHS Trust, Leicester, UK; 6 Department of Food Technology, Safety and Health, Ghent University, Coupure Links 653, Ghent, 9000 Belgium; 7 Department of Public and Occupational Health, Amsterdam UMC location VUmc, Vrije Universiteit Amsterdam, De Boelelaan 1117, Amsterdam, 1081 HV The Netherlands; 8 Flanders Institute for Healthy Living, G. Schildknechtstraat 9, Brussels, 1020 Belgium

**Keywords:** Co-creation, Stakeholder engagement, Youth, Sitting, Inactivity, Classroom, Programme

## Abstract

**Background:**

Adolescents’ high levels of school-related sedentary behaviour pose health risks, yet existing interventions have shown limited effectiveness. Participatory research, which engages adolescents and other partners in intervention development, holds promise by tailoring interventions to their needs and context, which may improve intervention implementation and effectiveness. This study conducted an evaluation of a participatory process aimed at developing and implementing an intervention addressing adolescents’ school-related sedentary behaviour and the implementation process of this intervention.

**Methods:**

Intervention Mapping guided the participatory process with 12 pupils from Grade 7 and three teachers from a secondary school in Flanders, Belgium. To develop the intervention, ten sessions with pupils and five with teachers were organised. Next, the intervention was implemented over six weeks. Experiences were captured from reflection forms completed by pupils (*n* = 34) and researchers (*n* = 28), four group interviews with pupils (*n* = 15) and two with teachers (*n* = 5), as well as questionnaires completed by 31 pupils and 8 teachers. Qualitative data were analysed using reflexive thematic analysis. Descriptive statistics were calculated from questionnaire data.

**Results:**

Pupils appreciated being engaged, however, integrating the participatory sessions with pupils within regular lessons challenged participation due to for instance time constraints. Teachers preferred a more efficient, less time-intensive engagement that still made them feel heard. Moreover, pupils and teachers mentioned in group interviews that the early engagement of a large group of pupils and teachers was necessary in participatory research to build support for the intervention. As only a small group of teachers was engaged in the participatory process, not all teachers supported and implemented the intervention. Nevertheless, questionnaire data showed that most pupils were exposed to the intervention.

**Conclusions:**

Broader engagement from pupils and teachers was suggested to improve support for the intervention and enhance the implementation of the intervention. A future study will determine the effectiveness of the intervention.

**Trial registration:**

This study was retrospectively registered at the Open Science Framework (DOI: 10.17605/OSF.IO/Y8GEJ; registered on July 1, 2024).

**Supplementary Information:**

The online version contains supplementary material available at 10.1186/s12889-026-27847-7.

## Background

The transition from primary to secondary education is a critical period for an increase in sedentary time [1, 2], with European adolescents spending, on average, over seven hours per day sedentary [3]. Schools contribute substantially to adolescents’ sedentary time with adolescents spending 63–80% of total school time [4–6], and 75% of class time [4], in a sedentary way. Beyond school hours, adolescents engage in high amounts of sitting for school-related tasks such as homework [7]. We defined school-related sedentary behaviour as sedentary behaviours occurring during school hours (e.g., sitting in the classroom during instructions, desk-based work, screen use for educational purposes, sitting during lunchtime) or outside of school hours but within the influence of the school (e.g., homework, studying) [8]. Sedentary behaviour is associated with adverse health outcomes among adolescents, including poorer cardiometabolic health, unfavourable body composition, reduced fitness, poorer mental health, and less favourable behavioural conduct [9]. Conversely, evidence suggests that active lessons, compared to more school-related sedentary behaviours, are associated with more favourable health and well-being outcomes among adolescents [10].

Therefore, interventions targeting school-related sedentary behaviour among adolescents are needed. However, evidence for their effectiveness remains limited and inconsistent. Two systematic reviews have evaluated classroom-based and school-based interventions specifically targeting sedentary behaviour in adolescents [11, 12]. McMichan and colleagues reported that most classroom-based interventions were based on one or more theoretical frameworks (i.e., self-determination theory, social cognitive theory, theory of planned behaviour, social learning theory, and/or theory of meanings of behaviour), were primarily educational in nature (e.g., pupils teaching on sedentary behaviour to peers) and demonstrated no statistically significant overall effects on sedentary behaviour [11]. Hynynen and colleagues identified four school-based interventions targeting sedentary behaviour among older adolescents, employing diverse strategies such as web-based programmes, self-monitoring, counselling provided to pupils, teachers, and parents, and multi-component school and family approaches [12]. However, only two of these studies reported theoretical underpinnings for the intervention (i.e., transtheoretical model and/or social cognitive theory) [12]. Moreover, only two studies reported significant favourable effects on the number of self-reported sedentary bouts of 30 min per day or self-reported sedentary time for boys [12].

Participatory research has been suggested as a promising approach to improve the effectiveness of such interventions [13]. Participatory research engages individuals in the research process who are not necessarily trained in conducting research but are part of or advocate for the groups that should benefit from the research [14]. Involving adolescents and other relevant partners in formulating research questions and identifying strategies to address them ensures that solutions are tailored to adolescents’ specific needs and context [15–17]. With regard to the development of interventions, this means that participatory research increases the relevance of interventions, which may potentially improve the feasibility and implementation of interventions [15, 17]. Moreover, engaging adolescents in research provides opportunities for their social, emotional, and cognitive development, and fosters their skills as agents of change [15–17].

Despite the need to target school-related sedentary behaviour and the potential of participatory research, a recent scoping review showed that no interventions have specifically addressed school-related sedentary behaviour among adolescents using a participatory approach [18]. In addition, school-based participatory research targeting physical activity often failed to involve key partners such as teachers, despite their crucial role in intervention implementation. Furthermore, only one study combined participatory research with a structured, theory- and evidence-based planning protocol. This is important, as participatory approaches with adolescents may otherwise result in an unstructured process of intervention development. To address these gaps, we developed an intervention targeting school-related sedentary behaviour among adolescents in Grades 7 and 8 (the first two years of secondary education, with adolescents typically aged 12–14), involving both pupils and teachers in the participatory process. The participatory process was guided by Intervention Mapping (a planning protocol) [19]. By providing a detailed account of this development process, this study responds to calls for greater methodological clarity in participatory research [20, 21] and offers insight into how planning protocols can be integrated in participatory research.

Participatory research is complex to evaluate because it involves both a participatory development process and an intervention that results from it [22]. Evaluating only intervention outcomes may overlook important information about how the participatory process influenced the relevance and implementation of the intervention. Process evaluations can help address this by examining key aspects such as engagement, shared decision making, quality and feasibility of the implementation, underlying mechanisms, and contextual influences [21, 23]. However, such evaluations remain limited in adolescent participatory research. Moreover, given the novelty of a participatory approach within the domain of school-related sedentary behaviour, evaluating both the participatory development process and the implementation of the intervention is essential to generate methodological insights that can inform future participatory intervention research in schools. Therefore, this study aimed to evaluate the participatory development process and the implementation process of the resulting intervention targeting school-related sedentary behaviour in adolescents within the first two years of secondary education.

## Methods

We recruited one secondary school in which an intervention, targeting adolescents’ school-related sedentary behaviour, was developed together with pupils and teachers. Only one school was recruited as this study was designed as a pilot study and because this enabled an intense collaboration with pupils and teachers within the school. To evaluate the participatory development process and the implementation of the intervention developed using a participatory approach, both qualitative and quantitative evaluation methods were used. The checklist presented by Leask and colleagues was used to describe the participatory process [22], the template for intervention description and replication (TIDieR) was used to describe the intervention [24], and the standards for reporting qualitative research (SRQR) checklist was used to report the qualitative research conducted in this study [25]. The checklists are available in additional file 1. The study adhered to the declaration of Helsinki and received ethical approval from the Ghent University Hospital ethics committee (B6702021000879). The study protocol was registered at the Open Science Framework in August 2024 (accessible via: https://osf.io/y8gej) [26].

### Participants

The participating school was recruited in August 2021 by contacting the principal of a secondary school who had previously expressed her willingness to participate in research regarding school-related sedentary behaviour. In school year 2021–2022, 601 pupils (64% males) were enrolled in this school. The average Flemish education poverty indicator (OKI) for this school in school year 2021–2022 was 1.49. The OKI is a number between zero and four that indicates the extent to which pupils meet four characteristics: home language being non-Dutch, low education level of the mother, receiving an education allowance, and living in a neighbourhood with high levels of school delay. School delay is defined as a situation where a pupil’s current grade level is lower than what it should be based on their year of birth and normal academic progression. A higher OKI corresponds to a higher level of poverty. The average OKI for all secondary schools in Flanders in school year 2021–2022 was 1.08. As we considered it important that the school shared control in the project and that the project was tailored to the participating school, the school decided how many and which classes would participate in the project. Three classes from Grades 7 and 8 (adolescents typically aged 12–14) were selected to be exposed to the intervention: one class of Grade 7 from the general education track (which provides a broad and more theoretical curriculum), one class of Grade 7 from the vocational education track (offering a curriculum that is more practical and job-specific), and one class of Grade 8 from the general education track. The intervention was developed together with a separate group of pupils and teachers, hereafter referred to as the ‘participatory groups’. The class of Grade 7 from the vocational education track, consisting of 12 pupils (8 boys and 4 girls), was appointed by the school as the participatory group representing the pupils as pupils in this track typically have more flexibility within their curriculum to accommodate extracurricular activities during regular school hours. The school management asked the three class teachers (all female) from the participating classes to join the participatory group representing the teachers. Furthermore, all pupils (*n* = 50) and teachers who were exposed to the intervention were eligible and were invited to participate in the process evaluation of the study. Informed consent to participate was obtained from the participants, and in case of the pupils also from the parents or legal guardians.

### Procedure

**Fig. 1:**
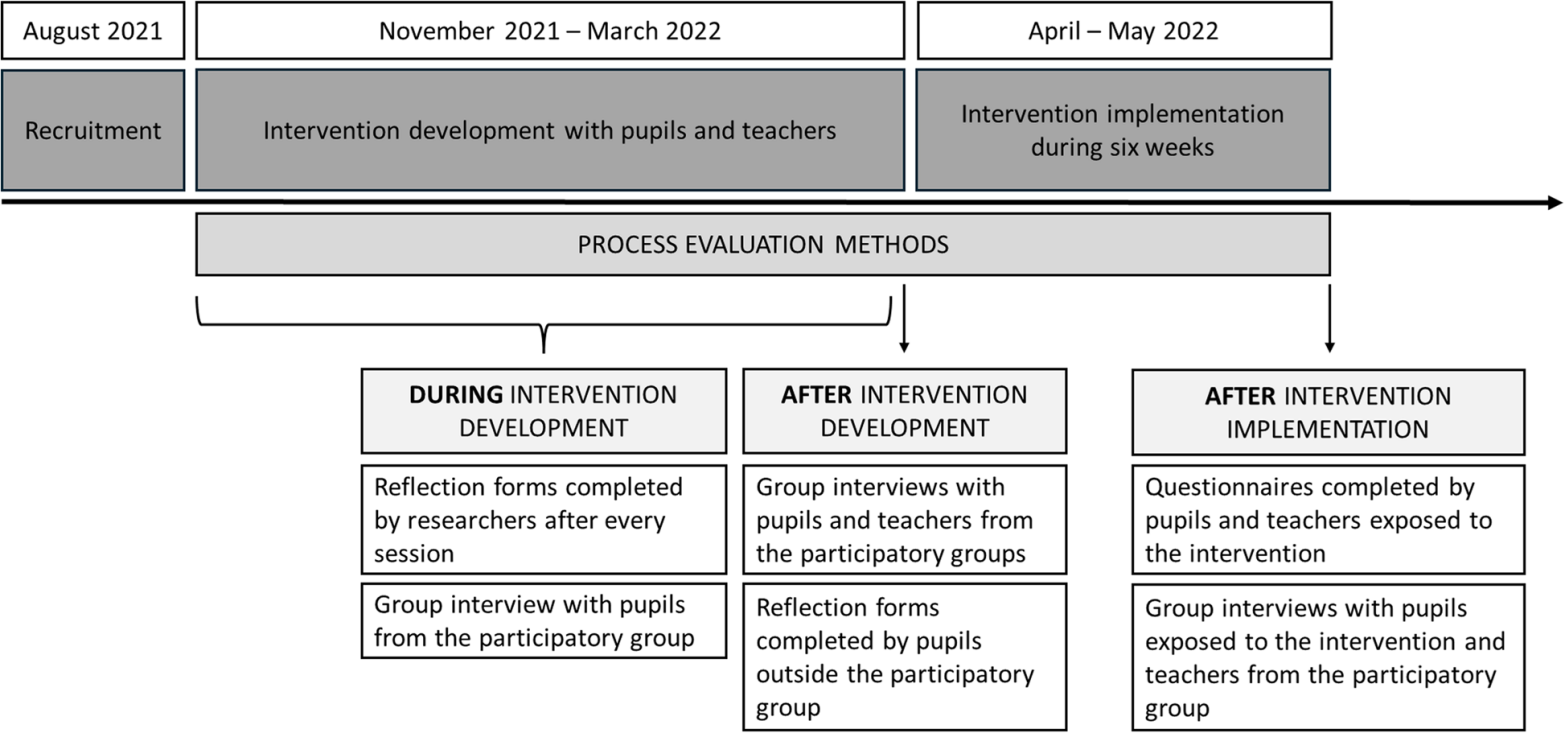
Overview of the study procedure and data collection for the evaluation of the participatory development process and the implementation of the intervention

The study procedure and data collection for the process evaluations are presented in Fig. 1. From November 2021 until March 2022, the intervention was developed together with the pupil and teacher participatory groups. While these participatory groups were primarily engaged in the participatory process, pupils from the other two classes and other teachers were also involved (see ‘the participatory process’). In April-May 2022, the intervention was implemented over six weeks.

Data for evaluating the participatory development process were collected through group interviews with pupils and teachers from the participatory groups. In addition, researchers completed a reflection form after each participatory session with the participatory group of pupils or teachers. Finally, pupils from classes not involved in the participatory group were invited to complete a reflection form about their involvement in developing the intervention.

To evaluate the implementation of the intervention, all pupils and teachers exposed to the intervention (including those of the participatory groups) were asked to complete a questionnaire after the intervention was implemented. In addition, two group interviews with pupils who were exposed to the intervention (including those of the participatory group) and one group interview with teachers from the participatory group were organised. The participatory process, the intervention and the data collection methods are described in more detail below.

### The participatory process

One researcher (VVO), supported by one to two colleagues facilitated the participatory sessions with both pupils and teachers. The participatory process with pupils included ten sessions of 50 min. The sessions were held during regular classes in the presence of the class teacher (who was also part of the teacher participatory group). With the participatory group of teachers, five online sessions of one hour each were organised after school hours. The participatory process was guided by Intervention Mapping, a structured six-step approach for developing effective, theory-based behaviour change interventions and placing significant emphasis on engaging relevant stakeholders throughout the process [19]. The six steps are: (1) conduct a needs assessment to identify what needs to be changed; (2) determine performance objectives (i.e., what does someone need to do to change the behaviour) and determinants (i.e., why would someone change the behaviour), and develop change objectives (i.e., what has to change in a determinant to achieve the performance objective); (3) select theory-based behaviour change methods to achieve the change objectives, and translate these into practical applications; (4) integrate the practical applications into an organised intervention programme and design materials; (5) plan for adoption, implementation, and sustainability of the programme; (6) generate an evaluation plan to conduct effect and process evaluations. The participatory group of pupils focused on steps 1–4 of Intervention Mapping. The participatory group of teachers reviewed the acceptability and feasibility of pupils’ ideas and suggested additional intervention strategies to improve the intervention. In addition, they created an implementation strategy for the intervention (step 5 of Intervention Mapping). The researchers conducted step 6 of Intervention Mapping. Although the participatory process primarily involved the participatory groups, we also engaged pupils from the two other participating classes. These pupils collaborated with the participatory group on a photovoice task exploring the causes of school-related sedentary behaviour. In addition, we organised a session in both classes in which we played a quiz to enhance pupils’ knowledge of sedentary behaviour, and during which pupils could provide feedback on the programme objectives drafted by the participatory group, and could brainstorm practical applications. A second session invited pupils to co-design or review the intervention strategies. Finally, pupils and teachers outside the participatory group took part in a competition to name the intervention. The participatory process and the integration of Intervention Mapping in the participatory process are explained in detail in additional file 2.

### The intervention

While the concept of school-related sedentary behaviour was approached broadly at the start of the participatory process, the target behaviour was refined to focus on reducing and regularly interrupting sedentary behaviour in two contexts: (1) during lessons at school (such as sitting during instructions and desk-based activities), and (2) during school-related activities at home (such as completing assignments or preparing for tests), as these were perceived as the most relevant contexts to target, while sedentary behaviour during school free time (e.g., recess) was not focused on. The participatory group developed an intervention, consisting of several intervention strategies directly targeting pupils’ sedentary behaviour, that would be implemented over six weeks, a duration that was determined to ensure feasibility within the pilot nature of the study (the intervention had to be developed, implemented, and evaluated within one school year).

In the first intervention week, a kick-off event was organised in which the school principal did some movement breaks with the pupils. In addition, a poster illustrating ways to monitor sedentary time was introduced to the pupils and each classroom was equipped with a kitchen timer. Finally, the first of three social media posts highlighting the benefits of reducing prolonged sitting was shared on the school’s social media channels, with subsequent posts in weeks 3 and 5. In week 2, a poster announcing the participation of the class in the project was displayed on the classroom door to demonstrate public commitment, and the first of five posters promoting the benefits of reducing prolonged sitting was displayed in the classroom and posted on the school’s online learning platform. A new poster was introduced in the classroom and on the school’s online learning platform every week. Additionally, pupils were shown two presentations with inspiration for movement breaks, and branded water bottles and pens were distributed to act as prompts to encourage pupils to reduce their sedentary behaviour and take regular breaks. Finally, every classroom was equipped with ten sit-to-stand desks, a name tag system so pupils and teachers could keep track of the pupils using the sit-to-stand desks, and a poster demonstrating the correct posture when using a sit-to-stand desk. During week 3, pupils were shown a video on how to create a standing desk at home, and a class competition was organised over several days to complete as many movement breaks as possible. In week 4, a class discussion on reducing and regularly interrupting sedentary behaviour during lessons at school and during school-related activities at home was held. Except for a new social media post and new posters on the benefits of reducing prolonged sitting, no new intervention strategies were introduced in weeks 5 and 6.

Intervention strategies were implemented by the researchers, by teachers from the participatory group (who were also the classroom teachers of the three participating classes), or by all teachers teaching in the three participating classes. To support the implementation, several implementation strategies (i.e., strategies to enhance the implementation of the intervention strategies targeting sedentary behaviour) were employed. First, a detailed implementation script was developed by the researchers. This script was also shared with the classroom teachers, who additionally received weekly emails from the researchers outlining upcoming tasks. For intervention strategies that required implementation by all teachers from the participating classes, a short, informal information session was organised during the lunch break prior to the start of the six-week implementation period. In addition, an email was sent to these teachers at the start of the implementation period specifying their tasks. Finally, the school’s learning platform manager and social media manager were responsible for sharing posters on the learning platform and the social media posts promoting the benefits of reducing prolonged sitting, respectively. Both received instructions via email detailing the implementation procedures. Next to the implementation strategies for teachers, a video was sent to the parents of the pupils being exposed to the intervention in the first intervention week to inform them about the project and to facilitate the implementation of the intervention at home. The implementation goal was that all intervention strategies (cfr. Table 1) would be implemented by all responsible implementers. A detailed explanation of the different intervention strategies, including the person(s) responsible for implementing each strategy, the behavioural determinants targeted among pupils, and the behaviour change techniques underlying each strategy, can be found in additional file 3.

**Table 1:** Overview of the different intervention strategies for pupils

WEEK 1	WEEK 2	WEEK 3	WEEK 4	WEEK 5	WEEK 6
Kick-off					
Poster illustrating ways to monitor sedentary time					
A kitchen timer was given to each class to optionally monitor sedentary time					
Social media post emphasising the benefits of sitting less		Social media post emphasising the benefits of sitting less		Social media post emphasising the benefits of sitting less	
	Poster on the classroom door announcing that the class was participating in the intervention				
	Poster in the classroom and on the school’s online learning platform emphasising the benefits of sitting less	Poster in the classroom and on the school’s online learning platform emphasising the benefits of sitting less	Poster in the classroom and on the school’s online learning platform emphasising the benefits of sitting less	Poster in the classroom and on the school’s online learning platform emphasising the benefits of sitting less	Poster in the classroom and on the school’s online learning platform emphasising the benefits of sitting less
	Pupils were given a water bottle featuring the intervention name and a pupil-written poem (highlighting the benefits of sitting less), and a pen with the intervention name and slogan				
	Two presentations to inspire pupils with movement breaks				
	Every classroom was equipped with ten sit-to-stand desks. The use of the sit-to-stand desks was supported by: → Name tag system so pupils and teachers could keep track of the pupils using the sit-to-stand desks → Poster showing the correct body posture when using a sit-to-stand desk				
		Class competition to complete as many movement breaks as possible			
		Video on how to create a standing desk at home			
			Class discussion on reducing and regularly interrupting sedentary behaviour during lessons at school and during school-related activities at home		

### Data collection

#### Evaluation of the participatory development process

A female researcher (VVO, MSc), trained in qualitative research, conducted a total of six group interviews—four with pupils and two with teachers (see Table 2). One or two additional researchers (JB and LRD, MSc) co-facilitated the group interviews and/or took notes. Pupils were invited face-to-face by their class teachers while participatory group teachers were invited face-to-face by the researchers to participate in the group interviews. At the start of each group interview, the interviewer outlined the goal. Next, a semi-structured group interview was conducted using an interview guide. Translated interview guides are available in additional file 4. Topics discussed during the group interviews were: motivation to engage in the participatory process, satisfaction with the participatory approach in general and with the organisation (e.g., frequency of sessions) and content (e.g., methods used) of the participatory sessions, engagement during the participatory sessions, collaboration with and support from others, feelings of control and ownership over the project, things learned, and sustaining pupil engagement at school. Group interviews lasted between 12 and 50 min, were audio recorded when conducted at school and video recorded when performed online, and transcribed verbatim.

**Table 2:** Overview of the group interviews conducted among pupils and teachers

Pupils or teachers (part of the participatory group or not)	When	Number of participants	Online or at school
Pupils (participatory group)	During intervention development (December 2021)	4 pupils (1 female)	Online
Pupils (participatory group)	After intervention development but before intervention implementation (March 2022)	3 pupils (2 female)	At school
Pupils (participatory group + non-participatory group)	After intervention implementation (June 2022)	5 pupils (4 female) *This group interview included 2 pupils from the participatory group (2 female) and 3 pupils outside the participatory group (2 female).*	At school
Pupils (non-participatory group)	After intervention implementation (June 2022)	3 pupils (2 female)	At school
Teachers (participatory group)	After intervention development but before intervention implementation (March 2022)	3 teachers (3 female)	Online
Teachers (participatory group)	After intervention implementation (June 2022)	2 teachers (2 female)	Online

To evaluate the perceptions of pupils not being part of the participatory group on their engagement in the participatory process, a form was delivered to these pupils after the development but before the implementation of the intervention, containing three open questions: (1) What did you like about the development of the intervention in the past months? (2) What did you not like about the development of the intervention in the past months? (3) Do you think you were able to express your opinion while designing the intervention? Why (not)?

Finally, at the end of each participatory session with pupils or teachers, the facilitating researchers completed a reflection form. The reflection forms were used to reflect on the group process (e.g., if everyone participated) and the role of the facilitators (e.g., if facilitators involved everyone) with the aim of improving the next session. A template of the reflection form can be found in additional file 5 [27].

#### Evaluation of the implementation process

The elements that were included in the process evaluation of the implementation of the intervention were based on the framework developed by Saunders and colleagues, entailing fidelity (i.e., the extent to which the intervention was implemented as planned including theoretical integrity and program quality), the dose delivered (i.e., the amount or number of intended units of each intervention or strategy delivered or provided by deliverers), the dose received (i.e., the extent to which participants actively engage with, interact with, are receptive to, and/or use materials), the satisfaction with the programme, and the context (i.e., aspects of the environment that may influence intervention implementation or study outcomes) [28]. Reach and recruitment were not assessed, as they were considered less relevant in the present study: the intervention was delivered during regular classroom hours and therefore reached the intended target group by design, and recruitment procedures at both the organisational and individual level were predefined and documented in the Methods section.

More specifically, these elements were evaluated as part of the group interviews that evaluated the participatory process. In addition, questionnaires were developed specifically for this study and administered to all pupils and teachers exposed to the intervention (including those of the participatory groups). Questionnaire development was guided by the conceptual definitions of three Saunders framework elements: dose delivered, dose received, and satisfaction. All items were pragmatically developed to align with the conceptual definition of the framework element and specific intervention strategies. Additional file 6 shows which items are linked to which process evaluation element. Fidelity and contextual factors were not assessed quantitatively, as these constructs are difficult to capture through questionnaires.

Finally, the first author contacted the school through an informal follow-up telephone call, approximately two years after the project ended (in March 2024), to ask whether and how the intervention was still being implemented. The school’s response was summarised by the researcher in a Word document during the call.

### Analyses

NVivo software (version 14.23.2) was employed for the analysis of group interview transcripts (*n* = 6) and reflection forms of pupils (*n* = 34) and researchers (*n* = 28, number of reflection forms per session ranged from one to three). A constructivist approach informed the analysis, acknowledging that reality is subjectively experienced and constructed [29]. This philosophical framework, which focuses on individuals’ subjective perceptions and the shared meanings they assign to a phenomenon—in this case, the participatory development process and (the implementation of) the intervention—was well aligned with our research aim of exploring pupils’ and teachers’ experiences with these processes [29]. The analysis followed Braun and Clarke’s six-phase reflexive thematic analysis method [30] and was conducted by the first author (VVO).

In reflexive thematic analysis, it is common for a single researcher to conduct the analysis, as themes are understood to be actively generated rather than “discovered” furthermore, the first author facilitated all participatory sessions and led the group interviews with pupils and teachers. [30]. The first author is a female PhD fellow with a background in physiotherapy and health promotion, and is affiliated with the Health Promotion unit at Ghent University. Her approach to participatory research was informed by both formal training and close collaboration with colleagues experienced in participatory research. In addition, she has extensive experience in working with adolescents during leisure time as a certified sports coach and has close personal relationships with individuals working as teachers, providing additional insight into their everyday realities. The researcher’s background and own experiences during the participatory process inevitably shaped the facilitation of the participatory process, and the collection and interpretation of the data. For example, her experience as a sports coach contributed to her confidence in facilitating the participatory sessions with pupils, while her awareness of teachers’ heavy workload led her to explicitly invite teachers to indicate when expectations became too demanding and to avoid assigning tasks to be completed outside the participatory sessions. These influences were acknowledged and reflected upon throughout the study.

In the first phase, VVO familiarised herself with the data by reading through the transcripts. In the second phase, initial codes were generated inductively. Phase three involved identifying broader patterns within the codes and developing preliminary themes. At this stage, VVO also made notes on potential relationships between themes. During the fourth phase, the themes were refined to ensure they accurately reflected the codes and overarching patterns in the data. During phase five, an initial thematic map was developed and subsequently reviewed by MV and BD to enrich the analysis through the exploration of multiple assumptions and interpretations. Next, VVO drafted a preliminary report. Furthermore, meaningful quotes were translated from Dutch to English by VVO. The final phase involved collecting feedback from all authors to finalise the analysis. In line with reflexive thematic analysis, data adequacy was not judged based on the concept of saturation, but rather on analytic sufficiency—that is, whether the dataset provided sufficiently rich, meaningful, and varied data to address the research questions [31]. The group interviews with pupils and teachers, and reflection forms from pupils and researchers were considered to be adequate to support the analyses. 

Descriptive statistics for the questionnaire data were calculated using IBM SPSS Statistics (version 29).

## Results

This section first presents the results based on the reflexive thematic analysis of the group interviews and reflection forms. This analysis entailed both elements of the evaluation of the participatory development process and the intervention implementation process. Next, this section presents the results of the questionnaires that evaluated the intervention implementation process. Finally, findings from the follow-up telephone call are presented.

### Process evaluation results from group interviews and reflection forms

**Fig. 2:**
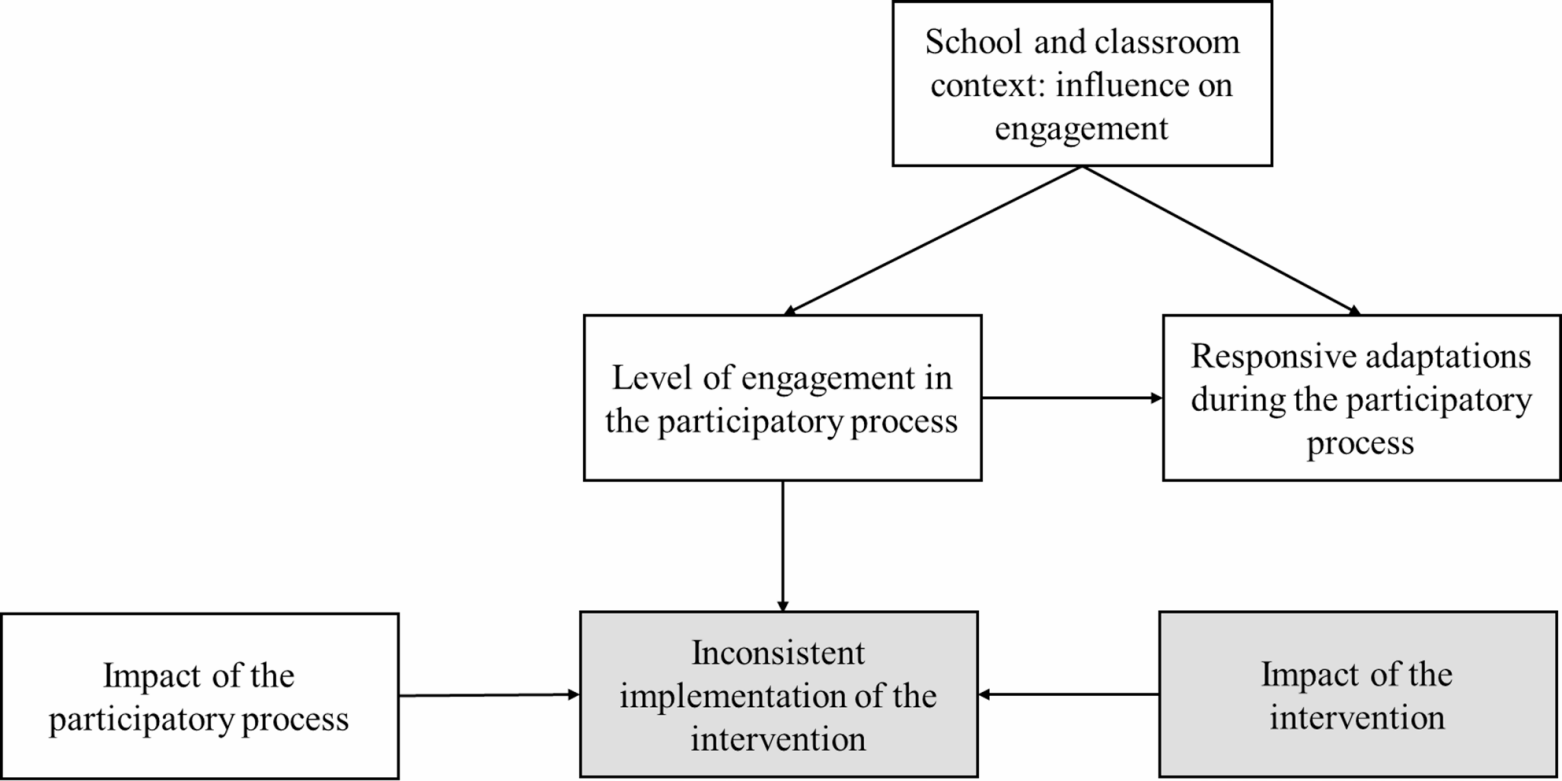
Thematic map showing the experiences of pupils and teachers with the participatory process and (the implementation of) the intervention. Note: Themes related to the evaluation of the participatory development process are shown in white boxes, while those related to the implementation of the intervention are shown in grey boxes

This section summarises the findings from the reflexive thematic analysis. Figure 2 presents the corresponding thematic map.

#### Themes related to the evaluation of the participatory development process

The evaluation of the participatory development process includes four themes. First, the school and classroom context shaped pupils’ and teachers’ engagement. For example, integrating the participatory process into regular lessons influenced researchers’ ability to distance the sessions from regular classroom practices and to engage all pupils similarly. Second, pupils and teachers differed in their preferred modes of engagement. Pupils preferred a playful mode of engagement and actively doing things during the sessions, such as creatively developing materials and activities themselves, while teachers preferred a more efficient participatory process, in which they could contribute ideas and feedback that were subsequently processed by the researchers. However, both groups emphasised the importance of early and broad engagement of pupils and teachers across the school in the participatory process. Third, maintaining engagement required ongoing responsive adaptations to the participatory process, with researchers, pupils, and teachers suggesting adaptations to the participatory process. Finally, participants reported perceived impacts of the participatory process, such as skill development, while the participatory process did not seem to have impacted their feelings of ownership of the project. Nevertheless, teachers in the participatory group felt responsible for implementing the intervention.

##### School and classroom context: influence on engagement

The school appointed the Grade 7 vocational education class as the participatory group because their curriculum offered the most opportunities for integrating the participatory process: *“That is also why we chose my class as the ‘pivotal figure’*,* as I would call it*,* to engage in the sessions*,* because that is where there is the most flexibility. (…) That is where the best opportunities are to do something outside of class.” [teacher 1*,* group interview after intervention development]*. At the same time, the researcher’s reflection forms revealed that integrating the participatory process into regular lessons was not straightforward. One researcher, for instance, stated: *“We are somewhat limited by the fact that we are working during a lesson. For example*,* we only have 50 minutes*,* which means we don’t have time to go to the playground for our warm-up*,* we have to make sure that we don’t disturb the other classes*,* and the class teacher remains present.” [researcher reflection form*,* session 4 with pupils]*. Such factors contributed to researchers struggling to break away from the school context during the participatory sessions: “*It is difficult to strike a balance between efficiency (due to time constraints) and ensuring that the context (in which the sessions are organised) is linked to school as little as possible.” [researcher reflection form*,* session 2 with pupils]*. This feeling was confirmed by some pupils who said they saw the researchers *“as a teacher*,* because you teach” [pupil 1*,* group interview after intervention development]*. Moreover, the researcher reflection forms showed that the limited time per session challenged the researchers to engage all pupils and teachers: *“I have tried to engage everyone as much as possible*,* but sometimes it is more difficult because of time constraints.” [researcher reflection form*,* session 6 with pupils].* Finally, as pupils were assigned by the school to engage in the participatory process, pupils noted that not everyone was motivated during the sessions: *“Yes*,* some children listened*,* but others were really preoccupied with other things.” [pupil 1*,* group interview after intervention development].* This was also evident in the researcher reflection forms: *“Most pupils participated. Others tried to cut corners by rushing through the assignments.” [researcher reflection form*,* session 8 with pupils]*.

##### Level of engagement in the participatory process

Pupils and teachers differed in how they preferred to engage during the participatory process. Pupils preferred active engagement. As one pupil said: *“I liked that you could really participate physically.” [pupil 2*,* group interview after intervention implementation]*. Teachers preferred a more efficient, low-effort form of engagement that still allowed them to feel heard: “*I felt that at that one hour*,* we worked well and there were a lot of ideas*,* that you guys as a team did a good job by taking them with you and working them out behind the scenes*,* which for me personally did make it really relaxing*,* because then all of a sudden there was such a ready-made package coming into the classroom.” [teacher 2*,* group interview after intervention development]*.

Furthermore, pupils and teachers emphasised the importance of early engagement from a wide group of pupils and teachers in participatory research. More specifically, pupils from the participatory group appreciated the engagement of pupils from outside the participatory group: *“I liked that other classes did that too (engaging in intervention development)*,* because then you could hear each other’s opinions.” [pupil 3*,* group interview after intervention implementation].* While pupils outside the participatory group liked that they were engaged, they would have preferred to be engaged earlier because *“we might have benefited more from it if we had done it at the beginning of the school year” [pupil 4*,* group interview after intervention implementation]*. Similarly, pupils and teachers mentioned that it might have been beneficial to engage more teachers from the outset: *“The colleagues involved in the project may also be briefed at the start*,* even if only one class continues with the participatory process.” [teacher 1*,* group interview after intervention implementation]*.

##### Responsive adaptations during the participatory process

To keep pupils and teachers engaged and to deal with the influence of the school and classroom context on pupils’ and teachers’ engagement, responsive adaptations were important throughout the participatory process. The researcher reflection forms showed that the researchers reflected on how they could improve the (next) participatory sessions, as illustrated in the following quote: *“Nevertheless*,* it was necessary to split up our group so that the girls could also express their opinions*,* as the boys were louder.” [researcher reflection form*,* session 3 with pupils]*. Or, another quote said: *“Some pupils are getting a little tired of “thinking”. We therefore have the idea of splitting the group into smaller groups from the next session onwards: the thinkers (who will continue with what we did during this session) and the doers (who will start developing the materials).” [researcher reflection form*,* session 5 with pupils]*. Moreover, the reflection forms showed that pupils and teachers also made suggestions for changes to the participatory process during the participatory sessions. For example, teachers proposed engaging all pupils in the participatory process rather than limiting it to the participatory group and suggested allocating two class hours to design intervention materials (e.g., drawing the posters) with pupils from two classes. During the intermediate group interview with pupils from the participatory group, pupils also mentioned things they would like to change about the participatory sessions. One pupil, for instance, said: *“I want to do a little more and talk less.” [pupil 5*,* group interview during intervention development]*. The group interviews also indicated that both pupils and teachers valued the integration of their suggestions. A pupil said, for instance, to feel listened to because: *“We said what we had written down (i.e.*,* a suggestion for future sessions) and we did that right away in the next session.” [pupil 6*,* group interview after intervention development]*. Finally, researchers also noticed positive effects on the group process when making responsive adaptations: *“The atmosphere was good*,* and in my opinion even better than in the previous sessions. I think they really enjoyed the game-like format and were therefore more motivated.” [researcher reflection form*,* session 6 with pupils]*. However, the researcher’s reflection forms also showed that researchers struggled to apply all suggested adaptations (like incorporating outdoor activities for the pupils). This was, for instance, due to the previously mentioned restrictions imposed by the school and classroom context (such as time constraints).

##### Impact of the participatory process

Pupils and teachers self-reported that participating in the participatory process raised awareness of sedentary behaviour among pupils and teachers in the participatory group. One pupil said: *“I didn’t really realise that we were sitting so much and stuff*,* because I didn’t know. I just didn’t pay attention to that.” [pupil 6*,* group interview after intervention development]*. Similarly, a teacher explained: *“I used to think*,* if they want to throw something in the bin*,* they can do that later. But now I think*,* no*,* let them stand up and walk to the bin and back.” [teacher 3*,* group interview after intervention implementation]*. Other pupils self-reported learning about collaborating with others: *“You learn that sometimes you need to speak up more.” [pupil 6*,* group interview after intervention development]*, or about developing a project: *“I learned how to use Canva.” [pupil 1*,* group interview after intervention development]*.

When pupils were asked whether they felt completely in charge of the project, pupils replied that they were in charge of this project *“but not without the teachers”*, and that *“you (the researchers) also helped”* to develop the intervention. The researcher’s reflection forms also reflected this sense of shared ownership, and even a lack of ownership of the project by the pupils: *“Although the pupils said they enjoyed it (the session)*,* they sometimes seem to lack motivation. They really need to feel that it is their project and they need to be fully committed to it.” [researcher reflection form*,* session 7 with pupils]*. Teachers in the participatory group did not want to take ownership of the project, as one teacher said, for example: *“I find that difficult because I don’t think we should take credit for this*,* as you have done everything. We just jumped on the bandwagon and pushed it along*,* but you did most of the work.” [teacher 1*,* group interview after intervention development].* Another teacher said: *“I think we are more ambassadors for the project rather than people who say*,* ‘look at what we are doing here’.” [teacher 3*,* group interview after intervention implementation]*. Nevertheless, teachers from the participatory group reported feeling responsible for successfully implementing the intervention: *“I think we are well prepared and know what is expected of us*,* and have been given all the tools to get on with it.” [teacher 3*,* group interview after intervention development]*. This was also confirmed by the researcher’s reflection forms: *“They also discussed together how they could convince their colleagues. The group feels responsible for this.” [researcher reflection form*,* session 3 with teachers]*.

#### Themes related to the implementation of the intervention

The evaluation of the intervention implementation included two themes. First, implementation varied across teachers, with teachers involved in the participatory process implementing the intervention more consistently compared to teachers outside the participatory group. Second, participants reported mixed perceptions regarding the impact of the intervention on classroom functioning, with some perceiving benefits for pupils’ learning, and others reporting disruptions to learning.

##### Inconsistent implementation of the intervention

Not all teachers outside the participatory group participated in implementing the intervention. One teacher from the participatory group mentioned for instance: *“I heard from a young colleague*,* who was so enthusiastic about doing a movement break every 20 minutes*,* that she often heard from the pupils that she was the only one doing it. She thought it was a pity that she was making so much effort while some other colleagues weren’t paying any attention to it.” [teacher 1*,* group interview after intervention implementation]*. Another teacher confirmed that *“younger colleagues tended to embrace this (the intervention) more openly than those from the slightly older generation” [teacher 3*,* group interview after intervention implementation]*. Similarly, a pupil said that *“most teachers do not implement (the intervention)”*. On the other hand, in the group interviews, pupils also mentioned several examples of teachers (including those outside the participatory group) who did implement the intervention. One pupil explained: *“During those games*,* we are actually learning. For example*,* if we have to tick something*,* the teacher says: ‘if you tick that box*,* then go and stand over there’. (…) In geography lessons*,* we are moving around anyway.” [pupil 7*,* group interview after intervention implementation]*. Another pupil gave the following example: *“With Mrs. X*,* instead of putting our heads down on the desk to relax*,* we now did a short (physical activity) break.” [pupil 8*,* group interview after intervention implementation]*.

Both pupils and teachers attributed the fact that not all teachers implemented the intervention to limited preparation of teachers outside the participatory group. In contrast, teachers in the participatory group reported feeling responsible for and capable of implementing the intervention. A teacher from the participatory group, for instance, said: *“I would also guess that this is something they thought came from outside*,* and perhaps they didn’t fully understand what the intention actually was*,* even though we tried to explain it to them.” [teacher 1*,* group interview after intervention implementation]*. Or, a pupil mentioned: *“I don’t know what goes on behind the scenes*,* but (I have a feeling) that you were more concerned with us than with the teachers.” [pupil 8*,* group interview after intervention implementation]*. As a result, pupils said that most teachers *“don’t know what to do” [pupil 8*,* group interview after intervention implementation]*, or that *“apart from teacher X*,* teachers don’t realise what we can do as a movement break*,* for example*” *[pupil 8*,* group interview after intervention implementation]*.

##### Impact of the intervention

Although not a primary intervention outcome, perceived effects on pupils’ learning were found to be important in determining teachers’ willingness to implement the intervention. Contrasting perceptions were reported regarding the perceived impact of the intervention on pupils’ learning in the classroom. Some participants perceived pupils’ concentration improved: *“And afterwards (i.e.*,* after going for a run on the playground)*,* they are really calm to continue.” [teacher 1*,* group interview after intervention implementation]*. Others perceived that standing disrupted pupils’ concentration or led to behavioural problems in the classroom: *“I just don’t like standing because I lose my concentration immediately.” [pupil 2*,* group interview after intervention implementation]*. Such unintended perceived negative effects also negatively influenced teachers’ willingness to implement the intervention, as this pupil mentioned: *“Maybe it’s our fault that those teachers don’t want this*,* so I think we need to start behaving ourselves.” [pupil 3*,* group interview after intervention implementation]*.

### Process evaluation results from the questionnaire

The descriptive statistics of the questionnaire data on (a) if and to what extent intervention strategies were implemented by teachers, and the exposure to and use of intervention strategies by pupils and teachers, (b) the interest in, attractiveness of, and liking of the strategies, and (c) the satisfaction with the participatory approach and the intervention in general are shown in additional file 7 and summarised below. A total of 31 pupils (62% male, mean age: 13.3 ± 0.7 years) and 8 teachers (13% male, mean age: 46.8 ± 10.5 years) completed the questionnaire.

Regarding the intervention dose delivered, results showed that 88% of the teachers implemented a movement break in at least half of their lessons during the six-week intervention period. None of the teachers implemented at least one movement break in every lesson. The number of movement breaks per lesson was higher during the class competition period with 75% of the teachers implementing at least one movement break per lesson at that time. Secondly, all teachers allowed pupils to use the sit-to-stand desks in every lesson. Furthermore, all five posters and all three posts on benefits of reducing prolonged sitting were implemented. Finally, the class discussions took place in all participating classes.

Regarding the dose received, most pupils (88%) reported exposure to movement breaks during the six-week intervention period, with most pupils (50%) performing one to four movement breaks per week. Related to the sit-to-stand desks, 77% of the pupils reported using them. Most pupils (46%) used them one to four times a week for an average of half an hour. Only 17% of the pupils reported using a standing desk at home. Furthermore, respectively only 30% and 7% of pupils recalled seeing all five posters and all three posts on benefits of reducing prolonged sitting. In addition, 63% of pupils used the intervention-branded water bottles and pens.

Teachers reported higher average scores regarding their interest in, the attractiveness of, and the liking of the intervention strategies compared to pupils. The average scores among pupils ranged from 2.9 ± 0.7 to 3.8 ± 1.0 and among teachers from 3.3 ± 0.6 to 4.7 ± 0.6 (items were scored on a 5-point scale with a higher score indicating a greater satisfaction). Similarly, satisfaction with the participatory approach and the intervention in general was observed to be rated higher by teachers than by pupils. Pupils’ average scores ranged between 3.5 ± 0.7 and 3.7 ± 0.8, while teachers’ scores ranged from 4.3 ± 0.9 to 5.0 ± 0.0 (except for the item on whether the intervention had an impact on pupils’ sedentary behaviour which had an average score of 3.5 ± 0.9).

### Maintenance of the intervention

During the follow-up telephone call, one participatory group teacher reported that sit-to-stand desks were still in use, with additional desks purchased for all Grade 7 and 8 classrooms. However, only a few teachers allowed their use and not all pupils were willing to use them when they were allowed to. In addition, all teachers were provided with sit-to-stand desks, although it was not mentioned whether all teachers used them. Furthermore, some teachers continued movement breaks, posters remained displayed, and photos of pupils engaging with the intervention were shared on the school’s social media. Finally, the teacher mentioned that the school management continued to support the implementation of the intervention.

## Discussion

The aim of this study was to evaluate a participatory process for developing an intervention addressing adolescents’ school-related sedentary behaviour as well as the implementation of this intervention. Pupils preferred active engagement during the participatory process, whereas teachers favoured a more efficient, low-effort approach that still made them feel heard. Furthermore, both pupils and teachers emphasised the importance of involving a large group of pupils and teachers from the outset of the participatory process. Finally, the intervention was largely implemented as planned by teachers who were engaged in the participatory process, however only a few teachers who did not engage in the participatory process allowed pupils to use sit-to-stand desks and performed movement breaks with the pupils.

### Evaluation frameworks

Before discussing the findings of this study, it is important to reflect on the process evaluation frameworks that informed our evaluation methods and on how this study advances process evaluations of school-based interventions developed using a participatory approach. For the process evaluation of the implementation of the intervention, we partly drew on the well-established framework by Saunders and colleagues [28]. However, for the evaluation of the participatory development process, no specific framework was available at the time the evaluation plan was designed. As traditional process evaluation frameworks, such as that of Saunders, have largely been developed in the context of top-down intervention development and implementation, these are limited in their ability to capture core elements of participatory research [21].

Recently, the PROSECO framework has been proposed to specifically support the evaluation of participatory development processes in public health interventions [32]. This framework comprises 37 components grouped into five dimensions: Delivery, Participation, Experiential, Context, and Impact [32]. Although PROSECO was not explicitly applied in the present study, a retrospective mapping indicates that all five dimensions were implicitly addressed in our evaluation. For example, Delivery was reflected upon in researcher reflection forms assessing the appropriateness of methods used during participatory sessions; Participation was explored through group interview questions about how pupils experienced working with their classmates; Experiential aspects were captured through group interview questions on experiences with the organisation, frequency, and content of the sessions; Context was addressed by questioning perceived support from the school environment in group interviews; and Impact was considered through reflections on capacity building among pupils and teachers in the researcher reflection forms. PROSECO encourages evaluators to selectively focus on the components within each dimension that are most relevant to their context and evaluation goals [32]. Therefore, a more deliberate selection and clearer articulation of the components under evaluation could strengthen future studies.

Importantly, while separate frameworks now exist for evaluating participatory processes (e.g., the PROSECO framework [32]) and the implementation of interventions (e.g., framework by Saunders and colleagues [28]), recent literature has called for greater integration of these frameworks [21]. By jointly evaluating the participatory development process and the intervention implementation process, and by explicitly examining how engagement during the participatory process influenced the subsequent implementation of the intervention, this study contributes to bridging frameworks evaluating participatory processes and frameworks evaluating the implementation of interventions.

### Engagement, ownership and responsibility

Our results showed that pupils and teachers differed in how they preferred to be involved in decision making during the participatory process. Pupils preferred a playful mode of engagement and actively doing things during the sessions, such as creatively developing intervention strategies themselves. Teachers, in contrast, preferred to contribute ideas and feedback that were subsequently processed by the researchers. Pupils engaged in the participatory process during class hours, which meant that the participatory process did not require extra time from them. In contrast, the participatory sessions with the teachers were held after school hours, making them an additional voluntary commitment on top of the high workload teachers experience [33]. A review on school staff experiences regarding their involvement in participatory processes also concluded that teachers prefer a guided participatory process in which high-level participation is less important because of their high workload [33]. Several participation models, such as Arnstein’s Ladder of Citizen Participation, present types of participation in a hierarchical structure, assuming that greater participant control and less researcher control are preferable [34]. Conversely, non-hierarchical models such as Treseder’s model [35], suggest that different situations require different types of participation, allowing participants to choose their level of engagement at different stages of the research process according to their availability and wishes [36]. Our findings are consistent with non-hierarchical, participatory models, demonstrating that the preferred levels and modes of engagement are shaped not only by the willingness to participate, but also by contextual factors, such as the timing of sessions.

Although the teachers were engaged at a lower level during the participatory process, they felt a greater sense of ownership over the project compared to pupils. This was reflected in their sense of responsibility for implementing the intervention. Several possible explanations may account for this. First, it is possible that the participating teachers were more motivated to engage in the participatory process than the pupils, as for teachers participation was voluntary whereas for pupils it was not. The fact that pupils were obliged to participate might also have reinforced existing power imbalances between pupils and teachers, and pupils and researchers, resulting in a reduced sense of ownership. Moreover, while the school management was eager to address school-related sedentary behaviour, pupils were not involved in selecting this topic. However, in participatory research, it is preferable that the issue of the participatory research project is perceived relevant and important by the target group [36, 37]. Although we tried to point out the relevance of targeting school-related sedentary behaviour in the first participatory sessions, it is possible that our way of working was not the most effective in achieving this. Future research should explore how to make a topic feel relevant to the target group. Secondly, teachers were engaged in both the development and implementation of the intervention, while pupils were only engaged in the development phase. Although teachers are crucial stakeholders in the implementation of school-based interventions [38], not engaging pupils in this phase resulted in pupils not having any responsibility during intervention implementation. This may have prevented pupils in the participatory group from feeling a sense of ownership over the project. Ideally, in participatory research, pupils are engaged in all stages of the research process, including the development, implementation, and evaluation of the intervention [22]. However, this also has its challenges due to time constraints during the participatory process and an increased participant burden—particularly when motivation is low to engage in the participatory process, as was the case for some pupils in this study. Third, the characteristics of the participatory group with pupils could provide an explanation (pupils from the vocational education track). In order to engage the adolescents during the sessions, the researchers had to ask very specific questions and provide examples to inspire them when requesting their input. This made the researchers reflect whether they were guiding the adolescents too much, thereby constraining power sharing—an essential principle of participatory research—and possibly affecting pupils’ perceived ownership of the intervention. Previous research has shown that participatory processes differ in schools offering general and technical education vs. schools offering technical and vocational education, with participatory research in technical or vocational education often requiring more structured guidance during the participatory process [38]. Finally, the results showed that researchers found it challenging to actively engage pupils and foster pupils' ownership, despite researchers’ efforts to differentiate the participatory sessions with pupils from traditional lessons by changing the classroom setup, using interactive methods to generate knowledge, and encouraging pupils to address the researchers by their first names. This could partly be attributed to the fact that the sessions were conducted during regular school hours within limited time slots, often with teachers present, which constrained opportunities to organise for instance sessions outside the classroom setting. As highlighted in a qualitative systematic review on factors influencing the implementation of youth participatory action research in high schools, integrating participatory projects into the school timetable can facilitate sustained participation and support participation from pupils who might otherwise not have engaged [39]. On the other hand, it may limit pupils’ ability to opt out and constrain youth autonomy by embedding the project within existing authority structures and norms [39]. As a result, pupils may perceive researchers as extensions of the school system rather than as external facilitators, which may influence how freely they express their views or steer the process more autonomously. When participatory research is conducted during instructional time, researchers should critically reflect on strategies to maximise opportunities for meaningful youth participation and project autonomy [39].

Next, our results showed that, although pupils from within and outside the participatory group appreciated being engaged, pupils outside the participatory group wanted to be engaged earlier in the development process. In addition, wider engagement of teachers outside the participatory group from the beginning would have been helpful to improve the tailoring of the intervention, thereby building support for the intervention among all teachers. Indeed, the results in our study showed that the intervention was positively received by teachers involved in the participatory process, who reported feeling responsible and capable of implementing it. However, uptake among teachers outside the participatory group was inconsistent due to a limited understanding of the intervention’s aims and strategies. This highlights the importance of engaging a broad range of participants early in the participatory process. However, as it is challenging to engage large groups at a high level of participation, different levels of involvement can be arranged within the same group of participants [17]. For example, a participatory research study aimed at promoting healthy sleep among adolescents engaged an adolescent participatory group as well as two pupils from each participating class, known as class coaches, who were engaged in (providing input for) the implementation of the intervention. Such class coaches could also serve as a bridge to share information with their classmates about the work done by the participatory group and then providing feedback to the participatory group in return [38].

### Implementation challenges

Although the participatory process was generally evaluated as positive, with the engagement from both pupils and teachers, a clear divergence emerged between the qualitative and quantitative findings regarding the implementation of the intervention by teachers. The group interview data suggested that only a small proportion of teachers outside the participatory group implemented the intervention, whereas the questionnaire data showed most teachers implemented movement breaks and allowed pupils to use the sit-to-stand desks in every lesson. This apparent inconsistency can be explained by the fact that only a convenience sample of eight teachers (including the three participatory group teachers) completed the questionnaire. It is therefore likely that these responses primarily reflect the experiences of teachers who were already supportive of the intervention and who implemented its strategies. In contrast, during the group interviews, the teachers who participated in the participatory process not only reflected on their own practices but also on the broader implementation within the school, including the behaviour of colleagues who were less involved or did not implement the intervention. As such, the interview data demonstrated the added value of qualitative data, especially for small convenience samples. Nevertheless, it would have been better if teachers who did not participate in the participatory process or did not implement the intervention had taken part in the group interviews.

To improve the implementation of interventions developed using a participatory approach, alongside the early engagement of a larger number of teachers as mentioned above, literature suggests that a formative evaluation during intervention implementation can be useful [21]. Our study proved successful in tailoring the participatory sessions to participants through formative evaluations during the participatory process using group interviews and researcher reflection forms. Such a formative evaluation during intervention implementation would have allowed for the identification and resolution of potential issues that hindered the intervention’s implementation [40].

### Maintenance of the intervention

An important aspect to consider in process evaluations of participatory developed health promoting interventions is maintenance, referring to the extent to which relationships and outcomes established during the participatory process or intervention are sustained over time [21]. In this study, maintenance was only minimally addressed during the participatory process. The time-intensive nature of participatory approaches [41] often limits the time available for implementing and embedding the intervention [42, 43], which should be considered in future research. Furthermore, apart from the informal follow-up telephone call, no formal follow-up data were collected in this study. Nevertheless, the insights obtained from this exploratory follow-up were encouraging. Although these exploratory findings do not allow firm conclusions about the maintenance of the intervention, literature suggests that one of the main benefits of participatory research is that interventions developed using a participatory approach are (more) likely to be sustained [15, 44]. Future research should therefore evaluate the maintenance of the participatory approach (i.e., continued youth engagement within the school) and developed strategies.

### Strengths and limitations

Importantly, the findings of this study should be interpreted in light of its primary aim, which was to evaluate the participatory development process and the implementation of the intervention developed using a participatory approach, rather than its behavioural effectiveness. While the process evaluation provides valuable insights into engagement, ownership, and implementation challenges, it does not allow conclusions to be drawn about the intervention’s effectiveness in reducing school-related sedentary behaviour. Evaluating behavioural outcomes requires an effectiveness study, which was beyond the scope of this manuscript.

Strengths of this study included that the participatory process was guided by the Intervention Mapping protocol, which is a rigorous stepwise approach for developing theory- and evidence-based health promoting interventions [19]. In addition, it is a strength that both pupils and teachers were engaged in the participatory process, and that efforts were made to engage all pupils in the participatory process increasing the likelihood that the intervention was acceptable to all pupils. Finally, the incorporation of perspectives from pupils, teachers, and researchers, using a variety of data collection methods ensured the conduct of a comprehensive evaluation of the participatory process and implementation of the intervention, although additional methods (e.g., observations during participatory sessions or during the implementation of the intervention) might have provided further insights.

A first limitation of this pilot study is that it was conducted in a single school recruited through convenience sampling, which limits the direct transferability of the intervention and process evaluation findings to other school contexts. At the same time, it is important to recognise that local embeddedness is an inherent characteristic of participatory research [45]. Interventions developed through participatory processes are shaped by the specific context and timing in which they are created, and are therefore not intended to be transferred as a standard package. Instead, interventions should be adapted to new settings, in line with for instance the cascade model in which interventions developed using a participatory approach are iteratively adapted when introduced in new contexts, taking into account contextual differences and their implications for implementation [22]. A further limitation concerns the relatively small samples participating in the evaluation of the implementation of the intervention. Thirty-one of the 50 pupils exposed to the intervention (62%) completed the process evaluation questionnaire, and eight of the 50 pupils (16%) participated in group interviews. Therefore, these findings may not fully represent the experiences of all pupils. Similarly, only a limited number of teachers completed the questionnaire and teacher group interviews on the implementation of the intervention included only participatory group teachers, making it likely that the perceptions of more actively involved teachers were overrepresented. Despite this limitation, the participating pupils and teachers also reflected on more critical issues and the perspectives of those who were less positive in the group interviews. Another limitation is that we did not conduct a member check to verify whether the conclusions drawn from the qualitative data accurately reflect the experiences of both pupils and teachers.

## Conclusions

Pupils and teachers preferred to engage differently in the participatory process, though both emphasised the importance of engaging a large group of pupils and teachers from the outset of the participatory process. However, as only a small group of teachers was engaged in the participatory process, not all teachers outside the participatory group supported and implemented the intervention. Nevertheless, most pupils were exposed to the intervention strategies. To increase support for the intervention and improve its implementation, future research could engage a broader range of pupils and teachers in the participatory process from the outset, offering different levels of participation. A future study will determine the effectiveness of the intervention developed using a participatory approach.

## Supplementary Information

Supplementary Material 1.

Supplementary Material 2.

Supplementary Material 3.

Supplementary Material 4.

Supplementary Material 5.

Supplementary Material 6.

Supplementary Material 7.

## Data Availability

Intervention materials are available upon request. The group interview transcripts and reflection forms analysed during the current study are not publicly available due to privacy concerns. However, the questionnaire data (collected in Dutch) are available from the corresponding author on reasonable request.

## Abbreviations

TIDieR: Template for Intervention Description and Replication
SRQR: Standards for Reporting Qualitative Research
OKI: Flemish education poverty indicator
