# Supplementary material for: Process evaluation of an intervention developed using a participatory approach targeting school-related sedentary behaviour among adolescents
Source: BMC Public Health. 2026 Jun 10;26:2368. doi: 10.1186/s12889-026-27847-7 (PMC13474841; doi:10.1186/s12889-026-27847-7)
Supplement: Supplementary file 1 [file 12889_2026_27847_MOESM1_ESM.docx]

**Additional file 1.1.** Checklist developed by Leask et al. (2019) for reporting the participatory process of intervention development.

| Section | Checklist item | |
| --- | --- | --- |
| Planning | | |
| How was the aim of the study framed? | 1) Use each element of the PRODUCES framework (PRoblem, Objective, Design, (end-) Users, Co-creators, Evaluation and Scalability) | Utilising youth participatory research (design) to develop (objective) - together with academic researchers, pupils, and teachers (co-creators) – an intervention to reduce and regularly interrupt school-related sedentary behaviour (problem) in adolescents (end-users). In this manuscript, a process evaluation of the participatory development process and implementation of the developed intervention were conducted (evaluation). The intervention was developed locally and within this study no specific scalability model was adopted. |
| Explain the sampling procedure | 2) Explain the criteria used for sampling | Cfr. ‘Participants’ |
|  | 3) In what settings did sampling occur? | Cfr. ‘Participants’ |
|  | 4) How many individuals engaged as co-creators (academic/non-academic stakeholders)? | Cfr. ‘Participants’, ‘The participatory process’ |
|  | 5) Describe the co-creators (demographics/groups/other characteristics of interest) | Cfr. ‘Participants’, ‘The participatory process’ |
| Conducting | | |
| How was ownership manifested? | 6) Explain the methods used to manifest ownership (for example, branding the group, identifying the rights and responsibilities of the group) | Participants were told of their equal status within the participatory group and their responsibility to contribute their ideas. |
| Procedure - Components: | 7) What level of participation was there from the co-creators? | Cfr. additional file 2 |
|  | 8) How was the overall aim presented? | The overall aim was explained at the start of the participatory process, and researchers indicated where each session fitted within the broader process throughout. |
|  | 9) How was the purpose of each meeting presented? | The purpose of each session was identified at the beginning of the session. |
|  | 10) What were the rules and responsibilities of participation agreed upon? | When establishing agreements with pupils, both the pupils and researchers agreed to be attentive, listen quietly and respectfully, avoid speaking over one another, and ensure everyone had the chance to share their views.  In discussions with teachers, they particularly stressed the importance of having a ‘listening ear’ to express their concerns about what they considered feasible and what not. |
| Procedure - Methods: | 11) In which areas did the co-creators require up-skilling? | Pupils and teachers were up-skilled regarding sedentary behaviour and intervention development. Academic researchers were upskilled regarding (1) pupils’ reasons for engaging in sedentary behaviour and preferences for reducing and regularly interrupting sedentary behaviour, and (2) teachers’ facilitators and barriers for implementing health promoting interventions. |
|  | 12) What previous evidence was reviewed, and how? | Contexts in which adolescents engage in sedentary behaviour and existing interventions targeting school-related sedentary behaviour. |
|  | 13) If a prototype was developed, describe the prototype and the prototyping process | Cfr. additional file 2. Most intervention strategies were initially prototyped by pupils or researchers. After this, the prototypes were provided with feedback by (other) pupils, after which the strategies were further refined. |
|  | 14) Describe the frequency and duration of meetings | Cfr. ‘The participatory process’, additional file 2 |
|  | 15) Give examples of interactive techniques or methods used | Cfr. additional file 2 |
|  | 16) Give examples of fieldwork techniques or methods used | Cfr. additional file 2 |
|  | 17) Give examples of how iteration occurred during the process | Cfr. additional file 2 |
| Evaluation | | |
| Process | 18) Explain how co-creator satisfaction and contribution were evaluated (for example reporting on attendance rates, questionnaires, interviews) | Cfr. ‘Procedure’, ‘Data collection’ |
|  | How are results reported back to stakeholders and the public? | Written reports will be developed and delivered to stakeholders. |
| Outcome | 19) Explain how the validity of the outcome and the process were evaluated (for example, face validation, member checking) | Member checking (i.e., co-creators reflecting on their previous discussions) and respondent validation (i.e., a summary of intervention strategies previously discussed are presented to co-creators and subsequently refined) occurred throughout the sessions (cfr. additional file 2). |
|  | 20) Explain plans for formal testing of the effectiveness/scalability of the co-created outcome | The intervention was embedded into a clustered controlled trial to assess the effectiveness of the intervention. |
|  | 21) Explain outcome of evaluation (if tested) | The outcomes evaluated include changes in sedentary time and time spent in sedentary bouts of ≥30 minutes (measured across the entire day, during class hours, outside class hours, and outside school hours), and changes in the determinants of reducing and regularly interrupting sedentary time. The effect evaluation of the intervention will be addressed in a separate manuscript. This manuscript focuses on the results of the process evaluation of the participatory approach and developed intervention. |

**Additional file 1.2.** TIDieR (Template for Intervention Description and Replication) checklist.

**The TIDieR (Template for Intervention Description and Replication) Checklist*:**

Information to include when describing an intervention and the location of the information.

| **Item number** | **Item** | **Where located **** | |
| --- | --- | --- | --- |
|  |  | Primary paper  ((sub)title where  the information is  mentioned) | Other ^†^ (details) |
|  | **BRIEF NAME** |  |  |
| **1.** | Provide the name or a phrase that describes the intervention. | ‘Background’ |  |
|  | **WHY** |  |  |
| **2.** | Describe any rationale, theory, or goal of the elements essential to the intervention. | ‘Background’ |  |
|  | **WHAT** |  |  |
| **3.** | Materials: Describe any physical or informational materials used in the intervention, including those provided to participants or used in intervention delivery or in training of intervention providers. Provide information on where the materials can be accessed (e.g., online appendix, URL). | ‘The intervention’ | The materials are available upon request. |
| **4.** | Procedures: Describe each of the procedures, activities, and/or processes used in the intervention, including any enabling or support activities. |  |  |
|  | **WHO PROVIDED** |  |  |
| **5.** | For each category of intervention provider (e.g., psychologist, nursing assistant), describe their expertise, background and any specific training given. | ‘The intervention’ |  |
|  | **HOW** |  |  |
| **6.** | Describe the modes of delivery (e.g., face-to-face or by some other mechanism, such as internet or telephone) of the intervention and whether it was provided individually or in a group. | ‘The intervention’ |  |
|  | **WHERE** |  |  |
| **7.** | Describe the type(s) of location(s) where the intervention occurred, including any necessary infrastructure or relevant features. | ‘Participants’ |  |
|  | **WHEN and HOW MUCH** |  |  |
| **8.** | Describe the number of times the intervention was delivered and over what period of time including the number of sessions, their schedule, and their duration, intensity or dose. | ‘Procedure’, ‘The intervention’ |  |
|  | **TAILORING** |  |  |
| **9.** | If the intervention was planned to be personalised, titrated or adapted, then describe what, why, when, and how. | ‘The participatory process’ |  |
|  | **MODIFICATIONS** |  |  |
| **10.^ǂ^** | If the intervention was modified during the course of the study, describe the changes (what, why, when, and how). | N.A. |  |
|  | **HOW WELL** |  |  |
| **11.** | Planned: If intervention adherence or fidelity was assessed, describe how and by whom, and if any strategies were used to maintain or improve fidelity, describe them. | ‘Data collection’ > ‘Evaluation of the implementation process’ |  |
| **12.^ǂ^** | Actual: If intervention adherence or fidelity was assessed, describe the extent to which the intervention was delivered as planned. | ‘Results’ |  |

** **Authors** - use N.A. if an item is not applicable for the intervention being described. **Reviewers** – use ‘?’ if information about the element is not reported/not sufficiently reported.

† If the information is not provided in the primary paper, give details of where this information is available. This may include locations such as a published protocol or other published papers (provide citation details) or a website (provide the URL).

ǂ If completing the TIDieR checklist for a protocol, these items are not relevant to the protocol and cannot be described until the study is complete.

* We strongly recommend using this checklist in conjunction with the TIDieR guide (see *BMJ* 2014;348:g1687) which contains an explanation and elaboration for each item.

* The focus of TIDieR is on reporting details of the intervention elements (and where relevant, comparison elements) of a study. Other elements and methodological features of studies are covered by other reporting statements and checklists and have not been duplicated as part of the TIDieR checklist. When a **randomised trial** is being reported, the TIDieR checklist should be used in conjunction with the CONSORT statement (see www.consort-statement.org) as an extension of **Item 5 of the CONSORT 2010 Statement.** When a **clinical trial** **protocol** is being reported, the TIDieR checklist should be used in conjunction with the SPIRIT statement as an extension of **Item 11 of the SPIRIT 2013 Statement** (see www.spirit-statement.org). For alternate study designs, TIDieR can be used in conjunction with the appropriate checklist for that study design (see www.equator-network.org).

**Additional file 1.3.** Standards for reporting qualitative research (SRQR) checklist.

|  |  | Reporting item | Reported under title… |
| --- | --- | --- | --- |
|  | #1 | Concise description of the nature and topic of the study identifying the study as qualitative or indicating the approach (e.g., ethnography, grounded theory) or data collection methods (e.g., interview, focus group) is recommended. | ‘Abstract’ |
|  | #2 | Summary of the key elements of the study using the abstract format of the intended publication; typically includes background, purpose, methods, results and conclusions. | ‘Abstract’ |
| Problem formulation | #3 | Description and significance of the problem/phenomenon studied: review of relevant theory and empirical work; problem statement. | ‘Background’ |
| Purpose or research question | #4 | Purpose of the study and specific objectives or questions. | ‘Background’ |
| Qualitative approach and research paradigm | #5 | Qualitative approach and guiding theory if appropriate; identifying the research paradigm is also recommended; rationale. The rationale should briefly discuss the justification for choosing that theory, approach, method or technique rather than other options available; the assumptions and limitations implicit in those choices and how those choices influence study conclusions and transferability. As appropriate the rationale for several items might be discussed together. | ‘Background’ (participatory research as guiding theory), ‘Analyses’ (reflexive thematic analysis as qualitative approach) |
| Researcher characteristics and reflexivity | #6 | Researchers' characteristics that may influence the research, including personal attributes, qualifications/experience, relationship with participants, assumptions and/or presuppositions; potential or actual interaction between researchers' characteristics and the research questions, approach, methods, results and/or transferability. | ‘Analyses’ |
| Context | #7 | Setting/site and salient contextual factors; rationale. | ‘Participants’ |
| Sampling strategy | #8 | How and why research participants, documents, or events were selected; criteria for deciding when no further sampling was necessary (e.g., sampling saturation); rationale. | ‘Participants’, ‘Data collection’ > ‘Evaluation of the participatory development process’ |
| Ethical issues pertaining to human subjects | #9 | Documentation of approval by an appropriate ethics review board and participant consent, or explanation for lack thereof; other confidentiality and data security issues. | ‘Methods’ |
| Data collection methods | #10 | Types of data collected; details of data collection procedures including (as appropriate) start and stop dates of data collection and analysis, iterative process, triangulation of sources/methods, and modification of procedures in response to evolving study findings; rationale. | ‘Procedure’, ‘Data collection’ |
| Data collection instruments and technologies | #11 | Description of instruments (e.g., interview guides, questionnaires) and devices (e.g., audio recorders) used for data collection; if/how the instruments(s) changed over the course of the study. | ‘Data collection’ |
| Units of study | #12 | Number and relevant characteristics of participants, documents, or events included in the study; level of participation (could be reported in results). | ‘Data collection’ > ‘Evaluation of the participatory development process’, ‘Analyses’ |
| Data processing | #13 | Methods for processing data prior to and during analysis, including transcription, data entry, data management and security, verification of data integrity, data coding, and anonymisation/deidentification of excerpts. | ‘Data collection’ > ‘Evaluation of the participatory development process’, ‘Analyses’ |
| Data analysis | #14 | Process by which inferences, themes, etc. were identified and developed, including the researchers involved in data analysis; usually references a specific paradigm or approach; rationale. | ‘Analyses’ |
| Techniques to enhance trustworthiness | #15 | Techniques to enhance trustworthiness and credibility of data analysis (e.g., member checking, audit trail, triangulation); rationale. | ‘Discussion’ |
| Syntheses and interpretation | #16 | Main findings (e.g., interpretations, inferences, and themes); might include development of a theory or model, or integration with prior research or theory. | ‘Results’ |
| Links to empirical data | #17 | Evidence (e.g., quotes, field notes, text excerpts, photographs) to substantiate analytic findings. | ‘Results’ |
| Intergration with prior work, implications, transferability and contribution(s) to the field | #18 | Short summary of main findings; explanation of how findings and conclusions connect to, support, elaborate on, or challenge conclusions of earlier scholarship; discussion of scope of application/generalizability; identification of unique contributions(s) to scholarship in a discipline or field. | ‘Discussion’ |
| Limitations | #19 | Trustworthiness and limitations of findings. | ‘Discussion’ |
| Conflicts of interest | #20 | Potential sources of influence of perceived influence on study conduct and conclusions; how these were managed. | None |
| Funding | #21 | Sources of funding and other support; role of funders in data collection, interpretation and reporting – no funding. | This work was supported by the Research Foundation Flanders (FWO) [grant number G072223N]. |
