# Supplementary material for: Process evaluation of an intervention developed using a participatory approach targeting school-related sedentary behaviour among adolescents
Source: BMC Public Health. 2026 Jun 10;26:2368. doi: 10.1186/s12889-026-27847-7 (PMC13474841; doi:10.1186/s12889-026-27847-7)
Supplement: Supplementary file 2 [file 12889_2026_27847_MOESM2_ESM.docx]

| **Additional file 2.** Chronological description of the participatory process and the integration of IM in the participatory process. | | | |
| --- | --- | --- | --- |
| **Who?**  **(Session No.)** | **Step IM** | **Aim** | **Method(s) used (including output resulting from the session)** |
| Pupils participatory group (1) | 1 | Increasing pupils’ knowledge of sedentary behaviour | Kahoot quiz |
|  |  | Exploring causes of sedentary behaviour | Pupils were asked to photograph aspects of their environment that caused them to sit down at school and during during school-related activities at home, to be shared in the next session (i.e., the photovoice method). |
| Pupils outside participatory group | 1 | **Exploring causes of sedentary behaviour** | Pupils from the other two classes participated in the photovoice task by taking photographs and submitting them to the researchers. |
| Pupils participatory group (2) | 1 | Exploring causes of sedentary behaviour | Researchers provided four large sheets for photos related to sedentary behaviour (1) in the classroom, (2) during recess/lunch, (3) during school-related activities at home, and (4) in other contexts. In group, pupils discussed each photo before placing them on the appropriate sheet (e.g., photo of a desk to make homework was placed on the sheet regarding school-related activities at home). Pupils also voted for the best photo per class as an extra fun activity. |
|  |  | Discussing the risk behaviour using questionnaire data | Before session 1, the researchers conducted a baseline measurement, including a questionnaire. During session 2, researchers showed histograms of the baseline questionnaire results of the items showing the time spent sedentary and the number of breaks in sedentary time in the classroom, during recess/lunch, and during school-related activities at home. A red minus was marked on a histogram if pupils and researchers assessed sedentary time was high or not regularly interrupted. Otherwise, a green plus was marked on the histogram. The histograms were then placed on the corresponding sheets with the photos. |
| Pupils participatory group (3) | 1, 2 | Summarising key problems | Pupils and researchers reviewed the photos and histograms on each sheet (from the previous session) to identify where sedentary time was high and/or not regularly interrupted. These conclusions were noted on the sheets. It was concluded that sedentary time was high and not regularly interrupted in the classroom and during school-related activities at home. |
|  |  | Formulating PO’s | The researchers provided an example how to break down a behavioural goal into subgoals (PO’s). Pupils then brainstormed in two groups—one focusing on reducing and interrupting sedentary behaviour in the classroom, and the other during school-related activities at home. Each group listed their PO’s on a designated sheet. Pupils developed three PO’s for reducing and regularly breaking up sedentary time in the classroom and three PO’s for reducing and regularly breaking up sedentary time during school-related activities at home (e.g., pupils follow lessons/do their school-related activities at home using standing desks). |
|  |  | Formulating CO’s | The researchers provided an example of how to link CO’s to a PO. Pupils then brainstormed in the same two groups on CO’s for each PO, and wrote down the CO’s on a designated sheet. The sheet guided them to think about whether they needed more knowledge, skills, a more favourable attitude, or a change in subjective norm to achieve a PO. An additional box allowed pupils to write down CO’s not covered by these determinants. The CO’s written down by the pupils reflected changes in knowledge, attitude, subjective norms, and skills (e.g., pupils express their ability to build a desk at home to do their school-related activities while standing). |
| Teachers participatory group (1) | 1, 2 | Increasing teachers’ knowledge of sedentary behaviour | Researchers gave a presentation on how much pupils sit, the adverse effects of prolonged sedentary behaviour, and sedentary behaviour guidelines. |
|  |  | Reviewing pupils’ needs assessment results | Teachers were shown the four sheets with photos and histograms on sedentary behaviour in various contexts. They were asked if they agreed with the pupils' conclusions and what additional insights they might draw from the needs assessment. Teachers acknowledged that school-related sedentary behaviour is an issue but believed that pupils outside the participatory group may not fully understand the need to change this behaviour. They also found it important that pupils realised that they sit frequently during non-school-related activities as well. To address these concerns, a session was planned for the two classes that were not part of the participatory group (see below). |
|  |  | Reviewing PO’s and CO’s drafted by the pupils | The PO’s and CO’s created by the pupils were shared, and teachers were asked to review them. Teachers were also asked if any objectives were missing. However, teachers agreed with the goals set by the pupils. |
| Teachers participatory group/  outside participatory group | N.A. | Announcing the project to teachers | Researchers distributed biscuits to all participating teachers, each with a card attached to announce the project. |
| Pupils participatory group (4) | 2, 3 | Reviewing PO’s and CO’s | Researchers displayed PO’s and CO’s from the previous session on a sheet. Each group of pupils reviewed and discussed the objectives set by the other group, noting similarities, differences, and potential additions to their own list. The PO related to regularly performing a movement break, which was listed to reduce and regularly interrupt sedentary time at home, was added to the PO’s aimed at reducing and regularly breaking up sedentary time in the classroom. |
|  |  | Brainstorming methods and applications | Researchers provided two examples of methods and applications for each determinant. Pupils were then divided into small groups (approximately two pupils per group). Each group received a sheet with a PO and corresponding CO’s. Groups noted possible applications for each CO on the sheet (e.g., making a poster so pupils know the correct posture when standing in the classroom), which was then passed around for additional input from other groups. After the session, researchers reviewed the ideas, removing non-relevant suggestions and merging similar ideas. This resulted in a final list of 30 applications: 13 related to the home environment and 17 to the school environment. |
| Pupils participatory group (5) | 3 | Determining which applications to develop further (part 1) | The researchers provided the pupils with a large sheet displaying the PO’s, CO’s, and the applications resulting from the previous session. In group, pupils voted on the applications. Each pupil had a red, green, and white card. Researchers presented each application, and pupils raised the green card for good applications, the red card for poor ones, and the white card for additional suggestions. Since not all applications were discussed during this session, the remaining ones were addressed in the next session. |
| Pupils outside participatory group (1) | 1-3 | Increasing pupils’ knowledge of sedentary behaviour | Kahoot quiz in small groups |
|  |  | Reviewing PO’s drafted by the pupils from the participatory group | Pupils evaluated the PO’s drafted by the pupils from the participatory group in small groups using designated sheets, marking them as good, good with comments, or not good. For PO’s with comments or deemed not good, pupils provided an explanation. Finally, pupils were asked if there were objectives missing. After the session, researchers reviewed the sheets completed by the pupils. The pupils generally agreed with the goals set by the pupils from the participatory group. |
|  |  | Brainstorming applications | Pupils were divided into six teams, which competed in pairs. Teams wrote each idea for an application on a card and indicated the related PO. Cards with applications were placed on a three-in-a-row game board, with teams scoring points by achieving three in a row. After the session, researchers reviewed the applications and incorporated new, relevant ideas—such as creating a video to inform parents and siblings about the intervention—into the next sessions with pupils of the participatory group for further discussion. |
| Pupils participatory group (6) | 3 | Determining which applications to develop further (part 2) | The class was divided into three groups, each playing a game similar to ‘Jungle Speed’. There were two piles of cards: one with CO’s and the other with applications. Each card had symbols on the back. The game started by turning over a CO card. Pupils then took turns flipping application cards until the symbols matched, indicating the application was relevant to the CO. The pupil who grabbed the totem first earned points. The group then decided if the application should be developed further. Movement break cards were also integrated into the game. The game resulted in pupils (refining and) including certain applications while excluding others. |
| Teachers participatory group (2) | 4, 5 | Reviewing applications suggested by pupils (part 1) | Researchers provided teachers with an online document listing the applications proposed by the pupils. Teachers reviewed each application and marked them as feasible or not feasible. Non-feasible applications were discussed. E.g., pupils suggested creating a video for parents to inform them about the intervention which teachers initially found unfeasible as this was a lot of work. However, when researchers offered to collaborate with pupils to make the video, teachers agreed. |
|  |  | Determining key actors, and PO’s and CO’s for intervention implementation | Google Jamboard was used to explore two questions: “Who contributes to implementing the intervention?” (i.e., defining deliverers), and “What should these individuals do to implement the intervention?” (i.e., defining PO’s for the deliverers). Another Google Jamboard showed the PO’s for implementation, and teachers were asked to brainstorm about CO’s. Teachers from the participatory group and outside the participatory group were defined as deliverers of the intervention. Teachers developed four PO’s focused on teachers informing themselves about the intervention, discussing the implementation with colleagues, implementing the intervention, and encouraging pupils’ enthusiasm. Additionally, teachers formulated CO’s related to knowledge, attitude, subjective norms, and self-efficacy (e.g., expressing to feel capable of implementing the intervention). |
| Pupils participatory group (7) | 4 | Designing materials (part 1) | Pupils played a goose board game in pairs or a group of three. Rolling the dice determined how many spaces they moved and which task they had to complete. There were five stations, each dedicated to developing a different material or refining a specific intervention strategies (e.g., creating a poster with methods to track sedentary time). One of the five stations involved brainstorming about a competition to give a name to the intervention. Researchers equipped each station with an information sheet and inspirational examples. Rolling a six required pupils to complete a movement break. |
| Pupils and teachers participatory group/outside participatory group | 4 | Determining the intervention name | Pupils and teachers had the opportunity to propose and vote on an intervention name. The intervention name elected was “bankzitters”. |
| Pupils participatory group (8) | 4 | Designing materials (part 2) | Pupils worked in pairs or a group of three. Rolling the dice determined which intervention materials pupils had to develop or which intervention strategy they had to refine (e.g., at one station pupils voted on the promotional materials for the intervention based on input from the previous session). One station also involved drafting a timeline for implementing the intervention strategies. Again, each station had an information sheet and inspirational examples. Moreover, two game elements were included in the session: At the start, each group estimated the total number of pips rolled by all groups during the session. Pupils recorded their own dice rolls on the blackboard. Additionally, the session featured a game where groups built a tower using ‘acrobats’ (similar to the ‘Tower of Pisa’ game) without letting it fall. Each group could add an acrobat to the tower if they rolled a number they had already rolled once. |
| Teachers participatory group (3) | 4, 5 | Reviewing applications suggested by pupils (part 2) | Researchers prepared practical questions about how to implement the materials developed by pupils, which were discussed with the teachers. E.g., when researchers asked for the best way to share the social media posts developed by pupils, teachers explained that the school’s Instagram and Facebook accounts could be used. |
|  |  | Discussing the intervention timeline | Researchers showed the timeline for implementing the intervention strategies to the teachers, who provided their feedback (e.g., they suggested moving the class competition to week 3 to allow for an initial familiarisation period during which movement breaks could be tested). |
|  |  | Brainstorming applications for implementation | In group, teachers brainstormed and decided on applications to achieve the CO’s for implementation. They decided to announce the intervention in the weekly staff newsletter, include it in the school calendar for six weeks, organise a lunch meeting to inform colleagues, and send an email to all teachers with detailed information on implementing the intervention. |
| Pupils participatory group (9) | 4 | Designing materials (part 3) | After pupils created a draft timeline in the previous session, which was adapted during session 3 with the participatory group of teachers, researchers presented a final version for pupils’ feedback. Pupils were then divided into three groups, each playing a different game with specific cues to complete tasks. These tasks involved (1) identifying delivery channels for the applications (e.g., pupils decided that the posters demonstrating the correct posture when standing would only be displayed in the classroom and not at home) and (2) providing feedback on several materials (e.g., on the poster showing ways to track sedentary time), after which researchers processed pupils’ feedback. |
| Pupils participatory group (10) | 4 | Designing materials (part 4) | Pupils were divided into five groups, each creating a different material. The following materials were developed: (1) a video for parents about the intervention, (2) posters and (3) social media posts on the benefits of reducing/regularly interrupting sedentary behaviour, (4) a poster on a good posture when standing, and (5) presentations with movement break ideas for in the classroom and at home. |
| Pupils Grade 7 class general education (2) |  |  |  |
| Pupils Grade 8 class (2) | 4 | Reviewing materials | Researchers presented the timeline for intervention implementation to the pupils. Intervention materials were then exposed in the classroom with post-its for pupils to provide feedback. Pupils walked around and noted what they liked, disliked, or wished to add to each material. Researchers reviewed pupils’ feedback and integrated relevant and feasible feedback into the intervention materials (e.g., music was added to the video on how to build a standing desk at home). |
| Teachers participatory group (4) | 4, 5 | Reviewing applications suggested by pupils (part 3) | Researchers presented an overview of the applications suggested by the pupils. Teachers and researchers discussed via which channel the materials would be delivered and implementation tasks were divided. For instance, it was decided that the video for parents with information about the project would be sent to parents, pupils, and teachers via the online learning platform of the school at the day of the kick-off, with one of the researchers delivering the content for this communication and one of the participatory group teachers forwarding this email to all parents, pupils, and teachers. |
|  |  | Reviewing applications for implementation | Researchers presented an overview of the applications and asked questions to refine the implementation plan. Teachers and researchers discussed via which channel the materials would be delivered and implementation tasks were divided. To announce the intervention in the weekly staff newsletter and to send an email to all teachers with detailed information on implementing the intervention (via the online learning platform of the school), researchers would draft the messages, and send them to a teacher from the participatory group, who would then forward them to the appropriate persons. The timing for this communication was also agreed upon. Additionally, one of the participatory group teachers would ensure the intervention was added to the school calendar for six weeks. |
| Teachers participatory group (5) | 5 | Discussing intervention maintenance | Researchers explained the different levels of programme maintenance (e.g., passage, routine, niche saturation). Teachers and researchers then discussed the necessary actions, timelines, and responsibilities for maintaining the programme. Teachers proposed continuing (1) the use of sit-to-stand desks by for example rotating the sit-to-stand desks across classes and (2) movement breaks by for example organising a class competition a few times per year. They proposed discussing these ideas further in an already existing workgroup at school. |

*Abbreviations: N.A. = not applicable, IM = intervention mapping, PO = performance objective, CO = change objective.*
