# Supplementary material for: Process evaluation of an intervention developed using a participatory approach targeting school-related sedentary behaviour among adolescents
Source: BMC Public Health. 2026 Jun 10;26:2368. doi: 10.1186/s12889-026-27847-7 (PMC13474841; doi:10.1186/s12889-026-27847-7)
Supplement: Supplementary file 3 [file 12889_2026_27847_MOESM3_ESM.docx]

| **Additional file 3.** Overview of the different intervention strategies. | | | | | |
| --- | --- | --- | --- | --- | --- |
| **Intervention strategy** | **Developed by…** | **Person responsible for implementation** | **Implemented during week…** | **Pupils’ behavioural determinants targeted** | **Underlying behaviour change technique(s)^b^** |
| At the intervention kick-off, the **school** **principal** **did several movement breaks together with the pupils**. The principal provided instructions and encouraged the pupils. | The idea originated from the participatory group of teachers. The movement breaks were chosen by the school principal. | Researchers prepared the kick-off (e.g., setting everything up). The school principal did several movement breaks together with the pupils during the kick-off. | 1 | Attitude | Direct experience |
|  |  |  |  | Self-efficacy, skills | *Behavioural practice/rehearsal*, verbal persuasion/exhortation, *instruction on how to perform a behaviour*, *demonstration of the behaviour*, *social support*, reinforcement |
|  |  |  |  | Perceived norm | Information about others’ approval |
| Two **posters** illustrated ways to **monitor sedentary time** (e.g., set an alarm on your smartphone, before you start your homework indicate on your homework when you will stand up, etc.). These posters were shown to the pupils during the intervention kick-off. | One poster was designed by pupils and later refined by the researchers, after which the pupils provided further feedback. The second poster, featuring the same information but with a different layout chosen by the pupils, was created by the researchers. | Researchers prepared the kick-off (e.g., setting everything up) and showed the pupils the posters during the kick-off. | 1 | Knowledge | Advance organisers, using imagery |
|  |  |  |  | Self-efficacy | *Instruction on how to perform a behaviour*, *action planning* |
| Each class was given a **kitchen timer** to optionally time sedentary periods. The timers were distributed during the intervention kick-off. | The idea originated from the participatory group of teachers. The timers were chosen and ordered by researchers. | Researchers distributed the kitchen timers during the kick-off. All teachers were invited to use the kitchen timers to time sedentary periods during lessons. | 1 | Self-efficacy, skills | *Prompts/cues*, facilitation |
| **Social media posts emphasising the benefits of sitting less** were shared on the school’s Instagram and Facebook pages. | Pupils | Researchers emailed one of the teachers from the participatory group, asking them to share the social media posts. The teacher forwarded the email to the person responsible for managing the school's social media accounts and publishing the posts. | 1, 3, 5 | Attitude, knowledge | Consciousness raising, persuasive communication, *information about health consequences* |
| A **poster** on the classroom door **announced that the class was participating** in the intervention. | Researchers | Researchers provided the poster to the teachers of the participatory group. Teachers of the participatory group were asked to display the poster on the classroom door.^a^ | 2 | Self-efficacy | Public commitment |
| **Posters promoting the benefits of sitting less** were displayed in the classroom and posted on the school’s online learning platform homepage. | Pupils | Researchers provided the posters to the teachers of the participatory group. The teachers of the participatory group displayed the posters in the classroom.  Researchers emailed one of the teachers from the participatory group, asking them to share the posters on the schools’ learning platform. The teacher forwarded the email to the person responsible for managing the platform. | 2-6 | Attitude, knowledge | Consciousness raising, persuasive communication, *information about health consequences*, repeated exposure |
| Two **presentations** inspired pupils with **movement breaks**: one offering ideas for use in the classroom, and the other suggesting movement breaks during homework. Each slide in the presentation featured a movement break. The presentations were shown to the pupils in their classroom. | Pupils | Researchers emailed the presentations to the teachers of the participatory group. The teachers of the participatory group were asked to show the presentations to the pupils.^a^ | 2 | Knowledge | Advance organisers |
|  |  |  |  | Self-efficacy, skills | *Instruction on how to perform a behaviour* |
| Every classroom was equipped with **ten sit-to-stand desks**. | The pupils decided on the inclusion of active desks, while the teachers selected the type (i.e., sit-to-stand desks). The researchers then ordered the sit-to-stand desks. | Researchers provided the schools with sit-to-stand desks. The teachers of the participatory group were tasked with installing them in the classrooms. All teachers were asked to encourage their use.^a^ | 2-6 | Self-efficacy, skills | *Prompts/cues*, facilitation |
| Pupils had **name cards**. On days when they used a sit-to-stand desk, they placed a name card in a box at the front of the classroom. A teacher of the participatory group emptied this box weekly and returned the cards to the pupils, allowing both teachers and pupils to track sit-to-stand desk usage over the previous week. | The idea originated from the participatory group of teachers. The name cards were created by researchers. | Researchers provided the schools with name cards and accompanying small boxes. The teachers of the participatory group were asked to put a box in each participating classroom, explain the system to the pupils, and empty the boxes weekly, returning the cards to the pupils.^a^ | 2-6 | Self-efficacy, skills | Monitoring of behaviours by others without feedback, *prompts/cues* |
| Two **posters** in the classroom demonstrated the **correct body posture when using a sit-to-stand desk**. | One poster was created by pupils. The second was drafted by pupils and finalised by researchers. | Researchers provided the posters to the teachers of the participatory group. The teachers of the participatory group displayed the posters in the classroom.^a^ | 2-6 | Knowledge | Advance organisers |
|  |  |  |  | Self-efficacy, skills | *Instruction on how to perform a behaviour*, *demonstration of the behaviour*, *prompts/cues* |
| Pupils were given a **water bottle** featuring the intervention name and a poem. The poem, also displayed on one of the posters, highlighted the benefits of sitting less. | The water bottle idea originated from the pupils. Researchers ordered them. | Researchers provided the bottles for the participatory group of teachers. The teachers of the participatory group were then asked to distribute the bottles.^a^ | 2 | Attitude, knowledge | Consciousness raising, persuasive communication, *information about health consequences*, repeated exposure |
|  |  |  |  | Self-efficacy | *Prompts/cues* |
| Each pupil received a **pen** with the intervention name and slogan. | The pen idea originated from the pupils. Researchers ordered them. | Researchers provided the pens for the participatory group of teachers, who were then asked to distribute the pens.^a^ | 2 | Self-efficacy | *Prompts/cues* |
| A **video** shown to pupils in the classroom guided pupils on **how to create a standing desk at home**, offering suggestions on materials to use, where to position the desk, etc. | Pupils provided input, after which the researchers created a video. The pupils then gave further feedback. | Researchers emailed the video to the teachers of the participatory group. The teachers of the participatory group were asked to show the video to the pupils. | 3 | Knowledge | Advance organisers, using imagery |
|  |  |  |  | Self-efficacy | *Instruction on how to perform a behaviour*, *demonstration of the behaviour* |
| The three participating classes held a **competition** over several days to **complete as many movement breaks as possible**. Teachers recorded the number of breaks on a score sheet. The winning class received a free period (instead of having classes) in which they played games and were treated to snacks and drinks. | The competition idea originated from the pupils. Researchers developed the score sheet, which was provided with feedback by the pupils. | Researchers put up the score sheets in the classroom. All teachers were asked to facilitate movement breaks in the classroom with their pupils. After the competition, the researchers collected the score sheets and informed the participatory group of teachers of the winner, who was then announced by one teacher to the other teachers and pupils. | 3 | Attitude | Direct experience |
|  |  |  |  | Self-efficacy, skills | Feedback, *social support*, reinforcement, provide contingent rewards, *action planning* |
|  |  |  |  | Perceived norm | Provide opportunities for social comparison, *social support* |
| **Class discussion** on reducing and regularly interrupting sedentary behaviour in the classroom and during homework. Key questions included:   - What is already working well? - What do you still find challenging? - Do you have tips for your peers?   Pupils first responded to these questions on a Google Jamboard, followed by a group discussion in class. | Pupils provided input, after which the researchers proposed a format for the class discussion. The pupils then offered feedback on this format. | Teachers of the participatory group were asked to facilitate the class discussion using the template that was provided by the researchers. | 4 | Self-efficacy | *Problem solving, instruction on how to perform a behaviour* |
|  |  |  |  | Perceived norm | Information about others’ approval, provide opportunities for social comparison, *social support* |

*^a^ Researchers supported teachers with implementing these intervention strategies as researchers were present in the classroom during the implementation of these strategies. ^b^ Behaviour change techniques not in italics are taken from the ‘Taxonomy of Behaviour Change Methods: An Intervention Mapping Approach’ (1); behaviour change techniques printed in italics are taken from the ‘Behaviour Change Technique Taxonomy (v1)’ by Michie and colleagues (2).*

**References**

1. Kok G, Gottlieb NH, Peters GJ, Mullen PD, Parcel GS, Ruiter RA, Fernández ME, Markham C, Bartholomew LK. A taxonomy of behaviour change methods: an Intervention Mapping approach. Health Psychol Rev. 2016 Sep;10(3):297-312. doi: 10.1080/17437199.2015.1077155. Epub 2015 Oct 15. PMID: 26262912; PMCID: PMC4975080.
2. Michie S, Richardson M, Johnston M, Abraham C, Francis J, Hardeman W, Eccles MP, Cane J, Wood CE. The behavior change technique taxonomy (v1) of 93 hierarchically clustered techniques: building an international consensus for the reporting of behavior change interventions. Ann Behav Med. 2013 Aug;46(1):81-95. doi: 10.1007/s12160-013-9486-6. PMID: 23512568.
