# Supplementary material for: Process evaluation of an intervention developed using a participatory approach targeting school-related sedentary behaviour among adolescents
Source: BMC Public Health. 2026 Jun 10;26:2368. doi: 10.1186/s12889-026-27847-7 (PMC13474841; doi:10.1186/s12889-026-27847-7)
Supplement: Supplementary file 4 [file 12889_2026_27847_MOESM4_ESM.docx]

**Additional file 4.1.** Topic guide for the group interview with the participatory group of pupils in December to evaluate the participatory process.

Veerle came to introduce the project to you at the beginning of the school year.

- What did you think of the project when it was introduced?
- How did you think the project would go?
- Is the project currently going as you expected? If not, what has gone differently?

Are the sessions as you expected?

- If the sessions went differently to what you expected, how did they differ? Are you positively/negatively surprised?

What do you think about the way we work (e.g., how often we meet, the length of the sessions, the size of the group(s) we work in)? Are there things you would prefer to do differently? If so, what?

What do you think of everyone's involvement during the sessions?

- To what extent do you think the entire class group is involved?
- To what extent can everyone express his/her opinion?
- How do you feel about the atmosphere during the sessions?

What is it like working with the class group?

Which session did you like best? Why?

- We have already used a variety of approaches to work with you during the project, such as taking photos and working in small groups. What did you like best? Why?

What do you like about the sessions?/Are there other things you like about the sessions?

- Why do you like that?

What makes you feel a session was successful at the end? Why?

What do you dislike about the sessions?/Are there any other things you dislike about the sessions?

- Why don't you like that?
- Could you think of a solution for this yourself?

What are you expecting from the upcoming sessions?

What have you already learned? (E.g., regarding sedentary behaviour, planning a project, general skills,...)

What do you expect to learn?

Do you feel that you are in control/directing the project yourself? Why is that, do you think? Do you like/dislike this? Why?

- To what extent do you feel that you are listened to (both by us and the group)?
- To what extent do you feel that we understand you?
- To what extent do you feel that your opinion is important?
- To what extent do you feel that your ideas determine the project?

Do you feel supported by the pupils of the other two classes? Why is that, do you think?

- Do the pupils from the other two classes sometimes ask you about the project? Do you sometimes talk to the pupils from the other two classes about the project? Why yes/no?
  - If yes, do you like/dislike this? Why?
  - If not, would you like/dislike this? Why?

Do you feel supported by the teachers? Do you feel supported by the school? Why is that, do you think?

Do you think we should involve certain people more effectively?

- If not, why not?
- If yes, who? Why those persons? In what way should we involve these persons?

Could you describe in one word (or one sentence) how you see us/what role you think we have?

What do you think of us (the facilitators)? You may honestly say what you think. What do you think we do well? What do you think we could do better? This can help us learn.

What do you expect from the project?

When will the project be successful for you? Why?

Are there any other things you want to say?

**Additional file 4.2.** Topic guide for the group interview with the participatory group of pupils in March to evaluate the participatory process.

**Bold questions: The statement is read aloud. Pupils then write their answers on small whiteboards and show them to the other pupils.**

*The italic questions are emoji questions.* *A variety of emojis are placed on the table. A question is asked and pupils take an emoji from the table that best represents their answer. They then discuss why they chose that emoji.*

*How do you like the project so far?*

- Why did you choose that emoji?
- Is the project going as you expected? If not, what did you expect the project to be like?
- Can you give an example of what is (not) going well?

Statements about our way of working:

- **How do you feel about the number of times we saw each other to prepare the project? (Too little, good or too much?)**
- **How do you feel about the duration of a session being one class hour? (Too little, good or too much?)**
- **We planned the entire project with your class. Did you find this group size too small, just right, or too big?**
- **Did you enjoy working with the class group? (Yes/No?)**
- **During the sessions, we sometimes worked in smaller groups and sometimes with the whole class. Which did you prefer?**

*What did you think of the sessions?*

- Why did you choose that emoji?
- Can you give an example of something you thought was good or fun during the sessions? (Why did you like this?)
- Can you give an example of something you didn't like during the sessions? (Why did you not like this?) How would you change this?

Statements about everyone's involvement during the sessions:

- **Did you feel that the entire class group was involved during the sessions? (Yes/no?)**
- **Was everyone able to express their views? (Yes/no?)**
- **Did you like the atmosphere during the sessions? (Yes/no?)**

Statements on capacity building:

- **Did you learn anything? E.g., regarding sedentary behaviour, planning a project, general skills,... (Yes/no?) Can you give an example of something you have learned?**
- **Are there things you would have liked to learn? (Yes/no?) What?**

Statements on ownership:

- **Do you feel that we listen to you? (Yes or no?)**
- **Do you feel that your classmates listen to you? (Yes/no?)**
- **Do you feel that we understand you? (Yes/no?)**
- **Do you feel that your opinion is important? (Yes/no?)**
- **Do you feel that your ideas determine the project? (Yes/no?)**
- **Are you proud of the project you came up with? (Yes/no?)**
- **Are you proud of the materials (e.g., videos, posters,...) you made for the project? (Yes/no?)**

Sub-questions relating to the above statements: Can you give an example of how you noticed this? Do you like/dislike this? Why?

Statements on the collaboration with the other classes and support from the environment:

- **Do pupils from the other two classes sometimes ask you about the project? (Yes/no?) (If yes, can you give an example? Do you like/dislike this? Why? If not, would you like/dislike this? Why?)**
- **Do you sometimes talk to pupils from the other two classes about the project? (Yes/no?) Why?**
- **Did you like that we also involved pupils from the other two classes (e.g., when taking photos)?**
  - **Did you like that the pupils from the other two classes also took photos of their sitting behaviour? (Yes/no?)**
  - **Did you like that the pupils from the other two classes were allowed to contribute ideas for the project? (Yes/No?)**
  - **Did you like that the pupils from the other class helped making materials? (Yes/no?)**
  - **Did you like that the pupils from the other class gave feedback on what you made? (Yes/no?)**
- **Do you feel supported by the teachers? (Yes/no?) Can you give an example of when you felt supported or unsupported by the teachers?**
- **Do you feel supported by the school? (Yes/No?) Can you give an example of when you felt supported or unsupported by the school**

*Take an emoji that describes what you think of us. You can honestly say what you think.*

- Why did you choose that emoji?
- What do you think we do well?
- What do you think we could do better?

*Take an emoji that reflects what you expect from the project.*

- Why did you choose that emoji?

When will the project be successful for you? Why?

Are there any other things you want to say?

**Additional file 4.3.** Topic guide for the group interview with the participatory group of teachers in March to evaluate the participatory process.

- Why did you decide at the beginning of the project to commit to this project? Did the motivation for your commitment change along the way? If so, in what way?
- What do you think about the way we work (e.g., how often we meet, the length of the sessions, the size of the group we work in)? Are there things you would prefer to do differently? If so, what?
- Do you feel you are in control of the project? Why is that, do you think? Do you like/dislike this? Why?
- To what extent do you feel you are listened to (both by us and the group)?
- To what extent do you feel that we understand you?
- To what extent do you feel that your opinion is important?
- To what extent do you feel that your ideas determine the project?
- Do you feel supported by the other teachers and school management? Why is that, do you think?
- Do colleagues sometimes ask you about the project? Do you sometimes talk to the other teachers about the project? Why (not)?
- When will the project be successful for you? Why?
- Do you think the project will be implemented well? Why do you think that?

**Additional file 4.4.** Topic guide for the group interview with pupils in June to evaluate the participatory approach and the implementation of the developed intervention.

**General questions about the intervention**

- Over the past six weeks, your school participated in the ‘bankzitters’ project. What did you think of...
  - ... the development of the project?
  - ... the content of the project?
  - ... the implementation of the project?

(*A variety of emojis are placed on the table. A question is asked and pupils take an emoji from the table that best represents their answer. They then discuss why they chose that emoji.*)

**Questions about the intervention strategies**

- The project was divided into several strategies. We are curious to know what you thought of these strategies. Here on the table, you see images of all the strategies. Choose the photos of the strategies that you (1) liked, (2) disliked, (3) do not recognise. Why did you choose these photos?

**Questions about the effect of the intervention**

We are also interested in whether the project influenced your sitting behaviour.

- Did the project help you to sit less?
  - Yes: In what way did the project help you? Was there a specific strategy that helped you with this?
  - No: Why do you think the project did not help? Why do you find it difficult to change your sitting behaviour?
- What could we have done differently to make the project more effective?
- What should we keep in the project in the future?
- Have you noticed any other changes or effects of the project (on pupils, teachers, or within the school)? If so, which ones?

**Questions about the participatory approach**

In this project, we experimented with a new way of working. We developed the project together with pupils and teachers. This was not solely the work of researchers. We chose this approach to make the project more appealing to pupils and teachers and easier to implement in schools.

- What do you think about the project being created by pupils and teachers?

(*A variety of emojis are placed on the table. A question is asked and pupils take an emoji from the table that best represents their answer. They then discuss why they chose that emoji.*)

- Did you feel like you could have a say in decisions? Why (not)? What did you think about your level of involvement?
- Do you feel that this project was tailored to your school (both in terms of content and the way it was implemented)? If so, how? If not, how could we improve it?

**Closing question**

- Is there anything else you would like to say?

**Additional file 4.5.** Topic guide for the group interview with teachers in June to evaluate the participatory approach and the implementation of the developed intervention.

**General questions about the intervention**

- Over the past six weeks, your school participated in the ‘bankzitters’ project. What did you think of...
  - ... the development of the project?
  - ... the content of the project?
  - ... the implementation of the project?
- What did your colleagues think of the project?

**Questions about the intervention strategies**

- The project was divided into several strategies. We are curious to know what you thought of these strategies. Which parts did you (1) like, (2) dislike, (3) don’t you recognise? Why did you choose these strategies? Do you think your colleagues would put certain strategies under a different category? If so, which strategies and why?

**Questions about the effect of the intervention**

We are also interested in whether the project influenced pupils’ sitting behaviour.

- Did the project help pupils to sit less?
  - Yes: In what way did the project help pupils? Was there a specific strategy that helped pupils with this?
  - No: Why do you think the project did not help? Why do pupils find it difficult to change their sitting behaviour?
- What could we have done differently to make the project more effective?
- What should we keep in the project in the future?
- Have you noticed any other changes or effects of the project (on pupils, teachers, or within the school)? If so, which ones?

**Questions about the participatory approach**

In this project, we experimented with a new way of working. We developed the project together with pupils and teachers. This was not solely the work of researchers. We chose this approach to make the project more appealing to pupils and teachers and easier to implement in schools.

- What do you think about the project being created by pupils and teachers?
- Did you feel like you could have a say in decisions? Why (not)? What did you think about your level of involvement?
- How did your colleagues, who were not in the participatory group, feel about their level of participation?
- Do you feel that this project was tailored to your school (both in terms of content and the way it was implemented)? If so, how? If not, how could we improve it?
- To what extent do you see the school and pupils working together to create things? To what extent do you see the school deciding things together with pupils? Are there any resources that could support this (e.g., a manual)?

**Closing question**

- Is there anything else you would like to say?
