# Supplementary material for: Process evaluation of an intervention developed using a participatory approach targeting school-related sedentary behaviour among adolescents
Source: BMC Public Health. 2026 Jun 10;26:2368. doi: 10.1186/s12889-026-27847-7 (PMC13474841; doi:10.1186/s12889-026-27847-7)
Supplement: Supplementary file 5 [file 12889_2026_27847_MOESM5_ESM.docx]

| **Additional file 6.** Questionnaire items evaluating the implementation of the intervention among pupils and teachers. | | | |
| --- | --- | --- | --- |
| **Questionnaire item** | **Variable name** | **Response options** | **Recoding** |
| **Pupils** | | | |
| **General questions** | | | |
| I am born as… | Sex | Boy/girl//I'd rather not say | N.A. |
| What is your date of birth? | Age | Open question | Based on the date of birth and the date the questionnaire was completed, the age of pupils in years was calculated. |
| **Exposure to the intervention strategies** | | | |
| During the first week, did you watch the video for parents with info about the project? This video was sent via Smartschool. | Exposure to video for parents | Yes/no/I did not receive the video | N.A. |
| Have you seen the videos with movement breaks to do at home and in the classroom? | Exposure to videos with movement breaks | Yes/no | N.A. |
| Have you used the videos with movement breaks to do at home and in the classroom? | Use of videos with movement breaks | Yes/no | N.A. |
| Have you seen the overviews of ways to keep track of your sedentary time? | Exposure to overviews of ways to keep track of sedentary time | Yes/no | N.A. |
| Have you used the overviews of ways to keep track of your sedentary time? | Use of overviews of ways to keep track of sedentary time | Yes/no | N.A. |
| Did you do movement breaks in class? | Exposure to movement breaks in class | Yes/no | N.A. |
| How many movement breaks did you do in class? | Number of movement breaks in class | Less than 1 per week/1 to 2 per week/3 to 4 per week/1 to 2 per school day/3 to 4 per school day/5 to 6 per school day/more than 6 per school day | Response options were grouped into four broader categories: less than 1 per week, 1 to 4 per week, 1 to 4 per day, 5 or more per day |
| Have you seen the poster showing the correct posture at a standing desk? | Exposure to poster on correct posture | Yes/no | N.A. |
| How many different posters (on the benefits of reducing prolonged sitting) have you seen on Smartschool and/or in the classroom? | Number of posters seen on benefits of reducing prolonged sitting | No/1/2/3/4/5 | N.A. |
| How many different posts (on the benefits of reducing prolonged sitting) have you seen on the Facebook or Instagram page of the school? | Number of posts seen on benefits of reducing prolonged sitting | No/1/2/3/I don't have a Facebook or Instagram account | N.A. |
| Did you use the branded water bottle? | Use of water bottle | Yes/no/I did not receive this | N.A. |
| Did you use the branded pen? | Use of pen | Yes/no/I did not receive this | N.A. |
| Have you used the sit-to-stand desks in your class? | Use of sit-to-stand desks in class | Yes/no | N.A. |
| How many times a week do you normally stand at a sit-to-stand desk in the classroom (Monday to Friday)? | How often the sit-to-stand desks were used in class | Less than once a week/once a week/2 times a week/3 times a week/4 times a week/  once a day/2 times a day/3 times a day/4 times a day/more than 4 times a day | Response options were grouped into four broader categories: less than once a week, 1 to 4 times/week, once a day, more than once a day. |
| On average, how many minutes do you normally stand at a sit-to-stand desk in class each time you use a sit-to-stand desk? | How long the sit-to-stand desks were used per turn in class | 0 to 5 minutes/6 to 10 minutes/11 to 15 minutes/16 to 20 minutes/21 to 25 minutes/26 to 30 minutes/31 to 35 minutes/36 to 40 minutes/41 to 45 minutes/46 to 50 minutes | Answer options were recoded to a number of minutes by using the midpoint method. So, for example, ‘0 to 5 minutes’ was converted to 2.5 minutes. |
| Did you put a name tag in the box when you used a sit-to-stand desk in the classroom? | Use of name tags | Yes/no/I did not get name tags | N.A. |
| Have you seen the video with info on building a standing desk at home? | Exposure to video on building a standing desk | Yes/no | N.A. |
| Did you use the video with info on building a standing desk at home? | Use of video on building a standing desk | Yes/no | N.A. |
| Did you use a standing desk during school-related activities at home? | Use of standing desk at home | Yes/no | N.A. |
| How many times a week do you normally stand at your standing desk at home (Monday to Sunday)? | How often the standing desks were used at home | Less than once a week/once a week/2 times a week/3 times a week/4 times a week/5 times a week/6 times a week/once a day/2 times a day/3 times a day/4 times a day/more than 4 times a day | Response options were grouped into four broader categories: less than once a week, 1 to 6 times/week, once a day, more than once a day. |
| On average, how many minutes do you normally stand at your standing desk at home each time you use your standing desk? | How long the standing desks were used per turn at home | 0 to 5 minutes/6 to 10 minutes/11 to 15 minutes/16 to 20 minutes/21 to 25 minutes/26 to 30 minutes/31 to 35 minutes/36 to 40 minutes/41 up to 45 minutes/46 up to 50 minutes/51 up to 55 minutes/56 to 60 minutes/more than 60 minutes | Answer options were recoded to a number of minutes by using the midpoint method. So, for example, ‘0 to 5 minutes’ was converted to 2.5 minutes. ‘More than 60 minutes’ was converted to 63 minutes. |
| In the fourth week, there was normally a class discussion about the project (in which, among other things, was discussed what had already gone well, what had gone less well and whether the pupils had any tips for each other). Did you take part in this class discussion? | Exposure to class discussion | Yes/no | N.A. |
| **Satisfaction with the intervention strategies** | | | |
| What did you think of the project in general? | Satisfaction with project in general | 1 = not nice at all/2 = not nice/3 = neutral/4 = nice/5 = very nice | N.A. |
| Did you find the below parts of the project interesting? | | | |
| Video for parents with info about the project | Interest in video for parents | 1 = not interesting at all/2 = not interesting/3 = neutral/4 = interesting/5 = very interesting | N.A. |
| Video with info on how to build a standing desk at home | Interest in video on building a standing desk |  | N.A. |
| Posters in the classroom and on Smartschool (showing benefits of reducing prolonged sitting) | Interest in posters on benefits of reducing prolonged sitting |  | N.A. |
| Posts on Instagram and Facebook (showing benefits of reducing prolonged sitting) | Interest in posts on benefits of reducing prolonged sitting |  | N.A. |
| Class discussion about the project. The discussion included what you are already doing well, what is still difficult and what tips you have for each other. | Interest in class discussion |  | N.A. |
| Did you find the below parts of the project appealing? | | | |
| Video for parents with info about the project | Attractiveness of video for parents | 1 = not attractive at all/2 = not attractive/3 = neutral/4 = attractive/5 = very attractive | N.A. |
| Video with info on how to build a standing desk at home | Attractiveness of video on building a standing desk |  | N.A. |
| Posters in the classroom and on Smartschool (showing benefits of reducing prolonged sitting) | Attractiveness of posters on benefits of reducing prolonged sitting |  | N.A. |
| Posts on Instagram and Facebook (showing benefits of reducing prolonged sitting) | Attractiveness of posts on benefits of reducing prolonged sitting |  | N.A. |
| Did you like the below parts of the project? | | | |
| Video for parents with info about the project | Liking the video for parents | 1 = not nice at all/2 = not nice/3 = neutral/4 = nice/5 = very nice | N.A. |
| Video with info on how to build a standing desk at home | Liking the video on building a standing desk |  | N.A. |
| Movement breaks | Liking the movement breaks |  | N.A. |
| Class competition with movement breaks | Liking the class competition |  | N.A. |
| Water bottles | Liking the water bottles |  | N.A. |
| Pens | Liking the pens |  | N.A. |
| Sit-to-stand desks in the classroom | Liking the sit-to-stand desks in the classroom |  | N.A. |
| Class discussion about the project. The discussion included what you are doing well, what is still difficult and what tips you have for each other. | Liking the class discussion |  | N.A. |
| **Satisfaction with participatory approach and intervention in general** | | | |
| I like that the project was made together with pupils from our school. | Idem as the questionnaire items | 1 = strongly disagree/2 = rather disagree/3 = neutral/4 = rather agree/5 = strongly agree | N.A. |
| I like that the project was developed especially for our class. |  |  | N.A. |
| I had a say in what the project would look like. |  |  | N.A. |
| I am proud of the say I had in the project. |  |  | N.A. |
| I think the project should be retained at school in the future. |  |  | N.A. |
| **Teachers** | | | |
| **General questions** | | | |
| What is your gender? | Gender | Male/female/X/I'd rather not say | N.A. |
| What is your age (in years)? | Age | Open question | N.A. |
| How many years of experience do you have as a teacher? | Years of teaching experience | Open question | N.A. |
| **Implementation of and exposure to the intervention strategies** | | | |
| During the first week, did you watch the video for parents with info about the project? This video was sent via Smartschool. | Exposure to video for parents | Yes/no/I did not receive the video | N.A. |
| Have you seen the videos with movement breaks to do at home and in the classroom? | Exposure to videos with movement breaks | Yes/no | N.A. |
| Have you used the videos with movement breaks to do at home and in the classroom? | Use of videos with movement breaks | Yes/no | N.A. |
| How often did you do movement breaks with pupils during your classes? | Number of movement breaks in class on regular days | I did several movement breaks every lesson/I did one movement break every lesson/I did more than half of the lessons a movement break/I did about half of the lessons a movement break/I did less than half of the lessons a movement break/I hardly ever did any movement breaks/ I didn’t do movement breaks | Response options were grouped into four broader categories: never, less than half of the lessons, at least half of the lessons, at least one movement break every lesson |
| How often did you do movement breaks with the pupils during the days when the class competition "for the most movement breaks" was organised? | Number of movement breaks in class during the class competition | I did several movement breaks every lesson/I did one movement break every lesson/I did more than half of the lessons a movement break/I did about half of the lessons a movement break/I did less than half of the lessons a movement break/I hardly ever did any movement breaks/ I didn’t do movement breaks | Response options were grouped into four broader categories: never, less than half of the lessons, at least half of the lessons, at least one movement break every lesson |
| Have you seen the poster showing the correct posture at a standing desk? | Exposure to poster on correct posture | Yes/no | N.A. |
| *How many different posters (showing the benefits of reducing prolonged sitting) were put up in the classroom?* | *Number of posters on benefits of reducing prolonged sitting implemented* | *No/1/2/3/4/5* | *N.A.* |
| How many different posters (on the benefits of reducing prolonged sitting) have you seen on Smartschool and/or in the classroom? | Number of posters seen on benefits of reducing prolonged sitting | No/1/2/3/4/5 | N.A. |
| How many different posts (on the benefits of reducing prolonged sitting) have you seen on the Facebook or Instagram page of the school? | Number of posts seen on benefits of reducing prolonged sitting | No/1/2/3/I don't have a Facebook or Instagram account | N.A. |
| Did you use the printed drinking bottle? | Use of drinking bottle | Yes/no/I did not receive this | N.A. |
| Did you use the printed pen? | Use of pen | Yes/no/I did not receive this | N.A. |
| During your lessons, how often were pupils allowed to use the sit-to-stand desks? | How often pupils were allowed to use the sit-to-stand desks in class | During every lesson/during more than half of my lessons/during about half of my lessons/during less than half of my lessons/during none of my lessons/in the classroom where I teach there are no sit-to-stand desks | N.A. |
| *Was the box with name tags emptied weekly?* | *Implementation of box with name tags* | *Yes/no* | *N.A.* |
| *Was the video with info on how to build a standing desk at home shown in class?* | *Implementation of video on building a standing desk* | *Yes/no* | *N.A.* |
| *Did you do a class discussion on the project?* | *Implementation of the class discussion* | *Yes/no* | *N.A.* |
| **Satisfaction with the intervention strategies** | | |  |
| What did you think of the project in general? | Satisfaction with the project in general | 1 = not nice at all/2 = not nice/3 = neutral/4 = nice/5 = very nice | N.A. |
| What did your colleagues think of the project in general? | Satisfaction of colleagues with the project in general |  | N.A. |
| Did you find the below parts of the project interesting? | | | |
| Video for parents with info about the project | Interest in video for parents | 1 = not interesting at all/2 = not interesting/3 = neutral/4 = interesting/5 = very interesting/6 = I cannot answer this question because I am not familiar with this strategy of the project | The response category ‘I cannot answer this question because I am not familiar with this strategy of the project’ was recoded as missing. |
| *Video with info on how to build a standing desk at home* | *Interest in video on building a standing desk* |  |  |
| Posters in the classroom and on Smartschool (showing benefits of reducing prolonged sitting) | Interest in posters on benefits of reducing prolonged sitting |  |  |
| Posts on Instagram and Facebook (with benefits of reducing prolonged sitting) | Interest in posts on benefits of reducing prolonged sitting |  |  |
| *Class discussion about the project* | *Interest in class discussion* |  |  |
| Did you find the below parts of the project appealing? | | | |
| Video for parents with info about the project | Attractiveness of video for parents | 1 = not attractive at all/2 = not attractive/3 = neutral/4 = attractive/5 = very attractive/ 6 = I cannot answer this question because I am not familiar with this strategy of the project | The response category ‘I cannot answer this question because I am not familiar with this strategy of the project’ was recoded as missing. |
| *Video with info on how to build a standing desk at home* | *Attractiveness of video on building a standing desk* |  |  |
| Posters in the classroom and on Smartschool (showing benefits of reducing prolonged sitting) | Attractiveness of posters on benefits of reducing prolonged sitting |  |  |
| Posts on Instagram and Facebook (with benefits of reducing prolonged sitting) | Attractiveness of posts on benefits of reducing prolonged sitting |  |  |
| Did you like the parts of the project below? | | | |
| Video for parents with info about the project | Liking the video for parents | 1 = not nice at all/2 = not nice/3 = neutral/4 = nice/5 = very nice/6 = I cannot answer this question because I am not familiar with this strategy of the project | The response category ‘I cannot answer this question because I am not familiar with this strategy of the project’ was recoded as missing. |
| *Video with info on how to build a standing desk at home* | *Liking the video on building a standing desk* |  |  |
| Movement breaks | Liking the movement breaks |  |  |
| Class competition with movement breaks | Liking the class competition |  |  |
| Drinking bottles | Liking the drinking bottles |  |  |
| Pens | Liking the pens |  |  |
| Sit-to-stand desks in the classroom | Liking the sit-to-stand desks in the classroom |  |  |
| *Class discussion about the project* | *Liking the class discussion* |  |  |
| **Satisfaction with participatory approach and intervention in general** | | | |
| I was aware that this project took place at school. | Idem as the questionnaire items | 1 = strongly disagree/2 = rather disagree/3 = neutral/4 = rather agree/5 = strongly agree | N.A. |
| It was clear what was expected of me during the 6 weeks the project ran. |  |  | N.A. |
| I knew who I could contact to get more info about the project. |  |  | N.A. |
| I see the importance of a project to reduce pupils' sitting behaviour at school. |  |  | N.A. |
| The project has had an impact on pupils' sitting behaviour. |  |  | N.A. |
| I like that the project was made together with pupils from our school. |  |  | N.A. |
| I like that the project was made together with teachers from our school. |  |  | N.A. |
| Teachers were sufficiently involved in developing the project. |  |  | N.A. |
| Teachers had a sufficient say in how the project would be implemented at school. |  |  | N.A. |
| I like that the project was developed specifically for our school. |  |  | N.A. |
| I felt that the project was tailor-made for our school. |  |  | N.A. |
| I felt it was feasible to implement the elaborated project. |  |  | N.A. |
| I think the project should be retained at school in the future. |  |  | N.A. |
| I plan to continue using project materials in the future. |  |  | N.A. |

*Note: Items in italic were only asked to the class teachers that were part of the participatory group as they were responsible for implementing the intervention strategy covered by the questionnaire item.*
