# Supplementary material for: Process evaluation of an intervention developed using a participatory approach targeting school-related sedentary behaviour among adolescents
Source: BMC Public Health. 2026 Jun 10;26:2368. doi: 10.1186/s12889-026-27847-7 (PMC13474841; doi:10.1186/s12889-026-27847-7)
Supplement: Supplementary file 6 [file 12889_2026_27847_MOESM6_ESM.docx]

**Additional file 5.** Reflection form for researchers.^1^

The first section of the reflection form consisted of ten items (statements or questions) related to the group process, while the second section focused on six items concerning the facilitator's role. Responses to statements were provided using a scale of --, -, 0, +, ++, with the option to add further details if needed. The open-ended questions addressed what aspects of the group process or facilitator's role were successful and how facilitators could enhance both the group process and their role in future sessions.

| **Reflection form** | |
| --- | --- |
| **Group process** | |
| The goal of the meeting was clear | (--, -,0, +, ++) |
| Everyone participated | (--, -,0, +, ++) |
| Everyone could give their opinion | (--, -,0, +, ++) |
| The atmosphere/vibe was good | (--, -,0, +, ++) |
| Creativity | (--, -,0, +, ++) |
| Capacity building: the children learned something new | (--, -,0, +, ++) |
| What went well (group level)? | Open question |
| Which qualities did arise within the group? | Open question |
| How can we promote the qualities within the group more? | Open question |
| What can be improved according to the group process? | Open question |
| **Role of the facilitator** | |
| Facilitators were clear | (--, -,0, +, ++) |
| Facilitators involved everyone | (--, -,0, +, ++) |
| Facilitators had a positive influence on the group atmosphere | (--, -,0, +, ++) |
| What went well (individual level)? | Open question |
| Which personal qualities arose during the meeting? | Open question |
| What can be improved according to the facilitator’s role? | Open question |
| What went well related to the methods used, and what went less well? | Open question |

**References**

1. Emke, H., Vandendriessche, A., Chinapaw, M., Deforche, B., Verloigne, M., Altenburg, T., & Anselma, M. (2024). Facilitating co-research: lessons learned from reflection forms within three participatory action research projects. *Health Research Policy and Systems*, *22*(1), 117.
