# Supplementary material for: Process evaluation of an intervention developed using a participatory approach targeting school-related sedentary behaviour among adolescents
Source: BMC Public Health. 2026 Jun 10;26:2368. doi: 10.1186/s12889-026-27847-7 (PMC13474841; doi:10.1186/s12889-026-27847-7)
Supplement: Supplementary file 7 [file 12889_2026_27847_MOESM7_ESM.docx]

| **Additional file 7.** Descriptive statistics of the questionnaire data from pupils and teachers on the process evaluation of the implementation of the intervention. | | | | |
| --- | --- | --- | --- | --- |
|  | **Pupils** | | **Teachers** | |
| **Variable** | **N valid** | **Response** | **N valid** | **Response** |
| **Demographics** | | | | |
| Sex (n (%) male) | 29 | 18 (62.1%) |  |  |
| Gender (n (%) male) |  |  | 8 | 1 (12.5%) |
| Age (mean ± SD) | 31 | 13.3 ± 0.7 | 8 | 46.8 ± 10.5 |
| Years of teaching experience (mean ± SD) |  |  | 8 | 22.6 ± 12.1 |
| **Implementation of and exposure to the intervention strategies** | | | | |
| Exposure to video for parents (n (%)) | 31 |  | 8 |  |
| Yes |  | 14 (45.2%) |  | 4 (50.0%) |
| No |  | 7 (22.6%) |  | 4 (50.0%) |
| I did not receive the video |  | 10 (32.3%) |  | 0 (0.0%) |
| Exposure to videos with movement breaks (n (%) yes) | 31 | 19 (61.3%) | 8 | 7 (87.5%) |
| Use of videos with movement breaks (n (%) yes) | 30 | 12 (40.0%) | 8 | 4 (50.0%) |
| Exposure to overviews of ways to keep track of sedentary time (n (%) yes) | 30 | 18 (60.0%) |  |  |
| Use of overviews of ways to keep track of sedentary time (n (%) yes) | 30 | 13 (43.3%) |  |  |
| Exposure to movement breaks in class (n (%) yes) | 30 | 26 (86.7%) |  |  |
| Number of movement breaks in class (n (%)) | 26 |  |  |  |
| Less than 1 per week |  | 0 (0.0%) |  |  |
| 1 to 4 per week |  | 13 (50.0%) |  |  |
| 1 to 4 per day |  | 9 (34.6%) |  |  |
| 5 or more per day |  | 4 (15.4%) |  |  |
| Number of movement breaks in class on regular days (n (%)) |  |  | 8 |  |
| Never |  |  |  | 0 (0.0%) |
| Less than half of the lessons |  |  |  | 1 (12.5%) |
| At least half of the lessons |  |  |  | 7 (87.5%) |
| At least one movement break every lesson |  |  |  | 0 (0.0%) |
| Number of movement breaks in class during the class competition (n (%)) |  |  | 8 |  |
| Never |  |  |  | 0 (0.0%) |
| Less than half of the lessons |  |  |  | 0 (0.0%) |
| At least half of the lessons |  |  |  | 2 (25.0%) |
| At least one movement break every lesson |  |  |  | 6 (75.0%) |
| Exposure to poster on correct posture (n (%) yes) | 30 | 22 (73.3%) | 8 | 8 (100.0%) |
| Number of posters on benefits of reducing prolonged sitting implemented (n (%)) |  |  | 3^a^ |  |
| No |  |  |  | 0 (0.0%) |
| 1 |  |  |  | 0 (0.0%) |
| 2 |  |  |  | 0 (0.0%) |
| 3 |  |  |  | 0 (0.0%) |
| 4 |  |  |  | 0 (0.0%) |
| 5 |  |  |  | 3 (100.0%) |
| Number of posters seen on benefits of reducing prolonged sitting (n (%)) | 30 |  | 8 |  |
| No |  | 3 (10.0%) |  | 0 (0.0%) |
| 1 |  | 2 (6.7%) |  | 0 (0.0%) |
| 2 |  | 4 (13.3%) |  | 0 (0.0%) |
| 3 |  | 9 (30.0%) |  | 3 (37.5%) |
| 4 |  | 3 (10.0%) |  | 0 (0.0%) |
| 5 |  | 9 (30.0%) |  | 5 (62.5%) |
| Number of posts seen on benefits of reducing prolonged sitting (n (%)) | 30 |  | 8 |  |
| No |  | 15 (50.0%) |  | 1 (12.5%) |
| 1 |  | 2 (6.7%) |  | 2 (25.0%) |
| 2 |  | 7 (23.3%) |  | 2 (25.0%) |
| 3 |  | 2 (6.7%) |  | 2 (25.0%) |
| I don’t have a Facebook or Instagram account |  | 4 (13.3%) |  | 1 (12.5%) |
| Use of water bottle (n (%) yes) | 30 |  | 8 |  |
| Yes |  | 19 (63.3%) |  | 7 (87.5%) |
| No |  | 9 (30.0%) |  | 1 (12.5%) |
| I did not receive this |  | 2 (6.7%) |  | 0 (0.0%) |
| Use of pen (n (%) yes) | 30 |  | 8 |  |
| Yes |  | 19 (63.3%) |  | 6 (75.0%) |
| No |  | 6 (20.0%) |  | 2 (25.0%) |
| I did not receive this |  | 5 (16.7%) |  | 0 (0.0%) |
| Use of sit-to-stand desks in class (n (%) yes) | 30 | 23 (76.7%) |  |  |
| How often the sit-to-stand desks were used in class (n (%)) | 26 |  |  |  |
| Less than once a week |  | 3 (11.5%) |  |  |
| 1 to 4 times/week |  | 12 (46.2%) |  |  |
| Once a day |  | 2 (7.7%) |  |  |
| More than once a day |  | 6 (23.1%) |  |  |
| How long the sit-to-stand desks were used per turn in class in minutes (mean ± SD) | 23 | 30.3 ± 17.8 |  |  |
| How often pupils were allowed to use the sit-to-stand desks in class (n (%)) |  |  | 8 |  |
| During none of my lessons |  |  |  | 0 (0.0%) |
| During less than half of my lessons |  |  |  | 0 (0.0%) |
| During about half of my lessons |  |  |  | 0 (0.0%) |
| During more than half of my lessons |  |  |  | 0 (0.0%) |
| During every lesson |  |  |  | 6 (75.0%) |
| In the classroom where I teach there are no sit-to-stand desks |  |  |  | 2 (25.0%) |
| Implementation of box with name tags (n (%) yes) |  |  | 3^a^ | 2 (66.6%) |
| Use of name tags (n (%)) | 23 |  |  |  |
| Yes |  | 14 (60.9%) |  |  |
| No |  | 8 (34.8%) |  |  |
| I did not get name tags |  | 1 (4.3%) |  |  |
| Implementation of video on building a standing desk (n (%) yes) |  |  | 3^a^ | 2 (66.6%) |
| Exposure to video on building a standing desk (n (%) yes) | 30 | 15 (50.0%) |  |  |
| Use of video on building a standing desk (n (%) yes) | 30 | 10 (33.3%) |  |  |
| Use of standing desk at home (n (%) yes) | 30 | 5 (16.7%) |  |  |
| How often the standing desks were used at home (n (%)) | 5 |  |  |  |
| Less than once a week |  | 2 (40.0%) |  |  |
| 1 to 6 times/week |  | 3 (60.0%) |  |  |
| Once a day |  | 0 (0.0%) |  |  |
| More than once a day |  | 0 (0.0%) |  |  |
| How long the standing desks were used per turn at home in minutes (mean ± SD) | 5 | 10.8 ± 10.6 |  |  |
| Implementation of the class discussion (n (%) yes) |  |  | 3^a^ | 3 (100.0%) |
| Exposure to class discussion (n (%) yes) | 30 | 11 (36.7%) |  |  |
| **Satisfaction with the intervention strategies (all items are scored on a scale from 1 to 5) (mean ± SD)** | | | | |
| Satisfaction with project in general | 30 | 3.7 ± 1.0 | 8 | 4.3 ± 0.5 |
| Satisfaction of colleagues with the project in general |  |  | 8 | 3.3 ± 0.5 |
| Interest in video for parents | 30 | 3.2 ± 0.9 | 6^b^ | 3.8 ± 0.4 |
| Interest in video on building a standing desk | 30 | 3.2 ± 0.9 | 3^a^ | 3.7 ± 0.6 |
| Interest in posters on benefits of reducing prolonged sitting | 30 | 3.4 ± 0.9 | 8 | 4.1 ± 0.6 |
| Interest in posts on benefits of reducing prolonged sitting | 30 | 2.9 ± 1.0 | 6^b^ | 4.3 ± 0.5 |
| Interest in class discussion | 30 | 3.1 ± 0.9 | 3^a^ | 4.7 ± 0.6 |
| Attractiveness of video for parents | 30 | 3.0 ± 0.9 | 5^b^ | 4.0 ± 0.7 |
| Attractiveness of video on building a standing desk | 30 | 2.9 ± 0.8 | 3^a^ | 3.3 ± 0.6 |
| Attractiveness of posters on benefits of reducing prolonged sitting | 30 | 3.2 ± 0.6 | 8 | 4.0 ± 0.5 |
| Attractiveness of posts on benefits of reducing prolonged sitting | 30 | 2.9 ± 0.7 | 6^b^ | 4.2 ± 0.4 |
| Liking the video for parents | 30 | 2.9 ± 0.6 | 5^b^ | 3.8 ± 0.5 |
| Liking the video on building a standing desk | 30 | 3.0 ± 0.9 | 3^a^ | 3.7 ± 0.6 |
| Liking the movement breaks | 30 | 3.8 ± 1.0 | 7^b^ | 4.4 ± 0.5 |
| Liking the class competition | 30 | 3.7 ± 1.1 | 8 | 4.1 ± 0.4 |
| Liking the water bottles | 30 | 3.7 ± 1.1 | 8 | 4.4 ± 0.5 |
| Liking the pens | 30 | 3.8 ± 1.0 | 6^b^ | 4.3 ± 0.5 |
| Liking the sit-to-stand desks in the classroom | 30 | 3.7 ± 1.0 | 7^b^ | 4.6 ± 0.5 |
| Liking the class discussion | 30 | 3.3 ± 0.8 | 3^a^ | 4.3 ± 0.6 |
| **Satisfaction with participatory approach and intervention in general (all items are scored on a scale from 1 to 5) (mean ± S.D.)** | | | | |
| I was aware that this project took place at school |  |  | 8 | 5.0 ± 0.0 |
| It was clear what was expected of me during the 6 weeks the project ran |  |  | 8 | 4.9 ± 0.4 |
| I knew who I could contact to get more info about the project |  |  | 8 | 5.0 ± 0.0 |
| I see the importance of a project to reduce pupils' sitting behaviour at school |  |  | 8 | 4.9 ± 0.4 |
| The project has had an impact on pupils' sitting behaviour |  |  | 8 | 3.5 ± 0.9 |
| I like that the project was made together with pupils from our school | 30 | 3.7 ± 0.8 | 8 | 4.9 ± 0.4 |
| I like that the project was made together with teachers from our school |  |  | 8 | 4.6 ± 0.7 |
| I like that the project was developed specifically for our school |  |  | 8 | 4.6 ± 0.5 |
| I like that the project was developed especially for our class | 30 | 3.6 ± 0.8 |  |  |
| I felt that the project was tailor-made for our school |  |  | 8 | 4.8 ± 0.5 |
| I felt it was feasible to implement the elaborated project |  |  | 8 | 4.5 ± 0.5 |
| I had a say in what the project would look like | 30 | 3.5 ± 0.7 |  |  |
| I am proud of the say I had in the project | 30 | 3.5 ± 0.7 |  |  |
| Teachers were sufficiently involved in developing the project |  |  | 8 | 4.6 ± 0.5 |
| Teachers had a sufficient say in how the project would be implemented at school |  |  | 8 | 4.5 ± 0.5 |
| I think the project should be retained at school in the future | 30 | 3.8 ± 0.9 | 8 | 4.3 ± 0.9 |
| I plan to continue using project materials in the future. |  |  | 8 | 4.3 ± 0.9 |

*Abbreviations: SD = standard deviation.*

*^a^ These items were only answered by three teachers as they were only asked to the class teachers that were part of the participatory group as they were responsible for implementing the intervention strategy covered by the questionnaire item.*

*^b^ The number of valid responses does not equal eight because one or a few teachers indicated that they could not answer the question because they were not familiar with this intervention strategy.*
